# Direct-write of free-form building blocks for artificial magnetic 3D lattices

**DOI:** 10.1038/s41598-018-24431-x

**Published:** 2018-04-18

**Authors:** Lukas Keller, Mohanad K. I. Al Mamoori, Jonathan Pieper, Christian Gspan, Irina Stockem, Christian Schröder, Sven Barth, Robert Winkler, Harald Plank, Merlin Pohlit, Jens Müller, Michael Huth

**Affiliations:** 1 Institute of Physics, Goethe University, Frankfurt am Main, Frankfurt, Germany; 2 Graz Centre for Electron Microscopy, Graz, Austria; 3 Department of Physics, Chemistry, and Biology (IFM), Linköping University, Linköping, Sweden; 4 Bielefeld Institute for Applied Materials Research, Bielefeld University of Applied Sciences, Bielefeld, Germany; 5 Institute of Materials Chemistry, Vienna University of Technology, Wien, Austria; 6 Institute for Electron Microscopy and Nanoanalysis, Graz University of Technology, Graz, Austria

## Abstract

By the fabrication of periodically arranged nanomagnetic systems it is possible to engineer novel physical properties by realizing artificial lattice geometries that are not accessible via natural crystallization or chemical synthesis. This has been accomplished with great success in two dimensions in the fields of artificial spin ice and magnetic logic devices, to name just two. Although first proposals have been made to advance into three dimensions (3D), established nanofabrication pathways based on electron beam lithography have not been adapted to obtain free-form 3D nanostructures. Here we demonstrate the direct-write fabrication of freestanding ferromagnetic 3D nano-architectures. By employing micro-Hall sensing, we have determined the magnetic stray field generated by our free-form structures in an externally applied magnetic field and we have performed micromagnetic and macro-spin simulations to deduce the spatial magnetization profiles in the structures and analyze their switching behavior. Furthermore we show that the magnetic 3D elements can be combined with other 3D elements of different chemical composition and intrinsic material properties.

## Introduction

Nanomagnetic structures are ubiquitous, as they form the basic functional elements in various applications, such as in magnetic storage and information processing, magnonics and spintronics, see e. g.^1–3^. Nanomagnetic structures are traditionally planar, but recent work is expanding nanomagnetism into three dimensions and it has been generally recognized that in three-dimensional nanomagnets complex magnetic configurations with unprecedented properties become possible, see^4^ for a recent review. In the narrower sense of magnetic information storage and processing, the advantages of extending the typically 2D structures into the third dimension for higher integration density have already been realized and have lead to developments such as the racetrack memory^5^. Further on, in so-called artificial spin ice systems^6–13^, that are currently subject of intensive research efforts, the actual limitation to lithographically defined 2D arrays of interacting ferromagnetic nano-islands prevents investigations of novel phases that can emerge from the more complex ground states of frustrated lattices in 3D. This is why first steps into 3D artificial spin ice systems are now being taken by combining multilayer techniques with sophisticated electron beam lithography (EBL)^14,15^. However, standard lithography techniques are intrinsically designed for 2D pattern formation and, consequently, they are barely suitable for the fabrication of free-form 3D nanostructures. Focused electron beam induced deposition (FEBID) follows a different approach to overcome this EBL-related limitation. It represents a highly flexible direct-write fabrication method which allows for excellent control in creating 3D structures very much like 3D printing on the nanometer scale. FEBID uses precursor gases which, being adsorbed on a surface, are dissociated in the focussed electron beam to form the deposit. In most cases the resulting structures are not simple phase-pure metals or oxides. Instead, the resulting nanostructures typically contain significant amounts of carbon, which is predominantly part of the precursor species. Also, FEBID is a highly complex process in which many parameters, such as electron beam energy and current, precursor flow and adsorption characteristics, precursor diffusion and beam steering strategy all influence the final deposit shape and the deposit’s composition^16–18^. However, intensive research over the last decade has pushed the capabilities of FEBID in two important areas. It is now possible to obtain fully metallic nanostructures of Fe, Co and FeCo-alloys^19^ and also of Au and Pt^20–24^. In addition, very recently the simulation-guided nano-manufacturing of 3D structures has matured to such a degree that even complex 3D objects can now be fabricated under controlled conditions^18^. For pillar-like Fe and Co structures this has recently been demonstrated in a detailed investigation of the sample composition^25^. The next important scientific development is now the synergistic combination of these two developments towards the realization of free-form magnetic 3D nanostructures^26,27^. Here, we demonstrate this next step by showing different examples of free-form 3D magnetic nano-architectures which represent basic building blocks for 3D lattices in which, e. g., magnetic frustration effects can be addressed. The structures have been directly written on a high-resolution micro-Hall sensor. We followed the magnetization switching behavior by measuring the associated magnetic stray field during external field sweeps using a sensor with dimensions adapted to the size of the magnetic structures. With the help of micromagnetic and macro-spin model simulations we are able to reproduce the generated magnetic stray fields associated with the complex switching behavior. We furthermore demonstrate the successful fabrication of a 3D lattice using one of the building blocks and show how a mixing of ferromagnetic and non-magnetic sub-elements in the building blocks can be achieved. This latter point may become of considerable importance with regard to reducing the complexity in the magnetic switching behavior of the building blocks used in artificial 3D spin ice.

## Results

### Geometry and microstructure

**Figure 1:**
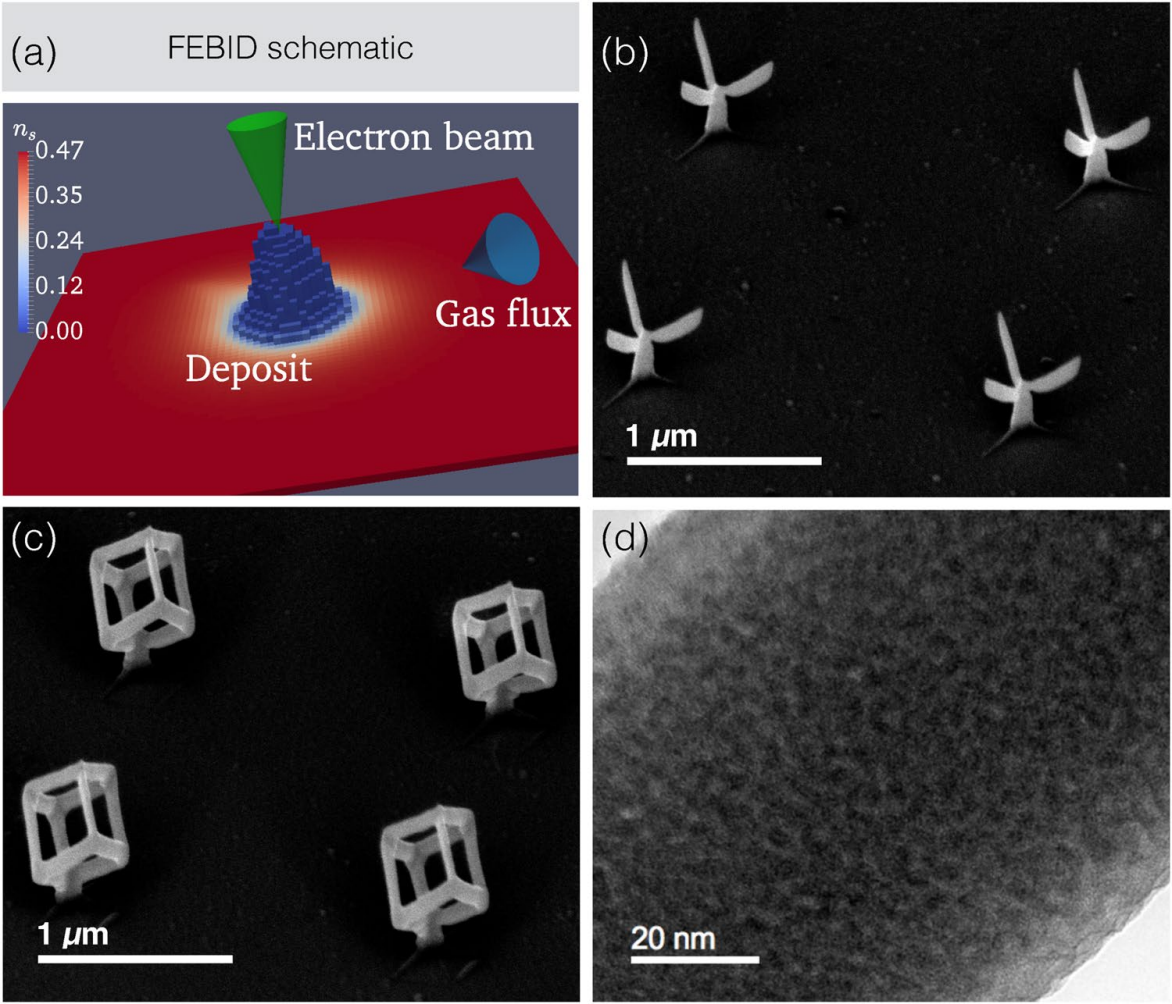
FEBID of 3D magnetic structures. (**a**) Schematic of FEBID process. *n*_*s*_ denotes the precursor coverage normalized to a maximum of one monolayer. See methods section for details. (**b**) and (**c**) SEM images of 2 × 2 arrays of Fe-Co nano-trees (**b**) and nano-cubes (**c**). The geometrical dimensions of the nano-trees are about 119 nm stem diameter (cylindrical), stem length 185 nm, branch/edge diameters 80 and 64 nm (roughly elliptical) and branch/edge length 340 nm. The geometrical dimensions of the nano-cubes are about 119 nm stem diameter (cylindrical), stem length 90 nm, edge diameters 94 and 75 nm (roughly elliptical) and edge length 340 nm. (**d**) Transmission electron microscopy bright field image of nano-cube region demonstrating the nano-granular microstructure.

For the deposition of magnetic 3D nanostructures by FEBID (see Fig. 1(a) for FEBID principle) we chose the recently introduced precursor HCo_3_Fe(CO)_12_, as this was shown in our previous work to yield deposits of high Co_3_Fe metal content under beam conditions which are suitable for writing high-resolution structures, i. e. high beam voltage and low beam current (see methods section for details)^28^. In view of very recent findings by Cordoba *et al*.^25^ it should be possible to obtain similar structures than the ones shown here with high metal content from the precursors Fe_2_(CO)_9_ and Co_2_(CO)_8_ under suitable beam conditions.

**Figure 2:**
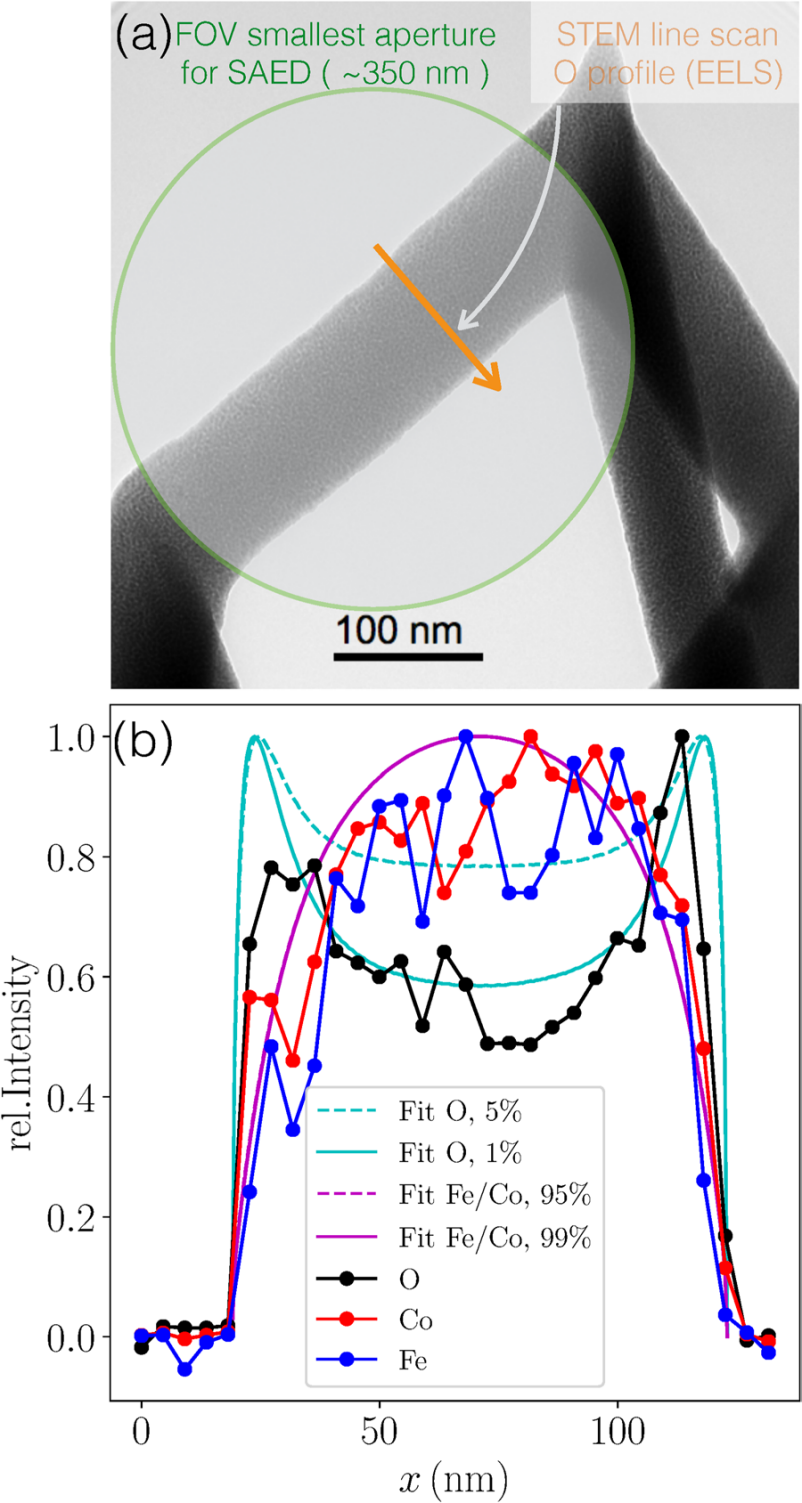
EELS line profiles. (**a**) TEM bright field image of an edge region of one nano-cube. The shaded area in the green circle indicates the region over which the selected area electron diffraction image, shown in Fig. 3 was taken (FOV: field of view). The orange arrow indicates the position and direction of the scanning TEM line along which EELS intensity data at the oxygen, iron and cobalt absorption energy were taken. (**b**) EELS intensity vs. scanning TEM line as indicated in (**a**) (dots and lines). The additional lines (solid and dashed) result from fits of an exponentially decaying (O) or increasing (Fe, Co) concentration from the surface to the center of the nano-cube edge, as detailed in the text. Note that the dashed and solid pink line almost perfectly coincide such that the dashed line is not separately visible. The fit parameters are: *R*_1_ = 50 nm (axis in scan(*x*) direction), *R*_2_ = 40 nm (axis in beam(*y*) direction), *λ* = 5.0 nm, ${f}_{max}^{(O)}={f}_{min}^{(Fe/Co)}\mathrm{=50}\,$ at%. The concentrations given in the legend refer to the assumed O, Fe and Co concentrations in the bulk, i. e. ${f}_{min}^{(O)}$ and ${f}_{max}^{(Fe/Co)}$.

In Fig. 1 we show two scanning electron microscopy (SEM) images taken directly after the writing of 2 × 2 arrays of Fe-Co nano-trees (b) and nano-cubes (c) onto the top Au gate of a GaAs/AlGaAs micro-Hall sensor (see methods section for details). These represent magnetic nanostructures with vertices from which three and four edges are emanating, respectively. In order to determine the microstructure and element distribution in the deposits we have performed transmission electron microscopy (TEM) experiments on nano-cubes grown onto metallic TEM grids. As the growth in 3D very sensitively depends on the precursor flux distribution^29^, special care was taken to reproduce the nano-cube geometry as obtained on the micro-Hall sensor. Microstructure and element distribution contain the essential information for developing an appropriate micromagnetic simulation model, as described later. In Fig. 1(d) we present a TEM bright field image of one of the nano-cube edges. The 3D edges reveal a homogeneously distributed nano-granular structure consisting of nano-crystallites of about (3 ± 0.5) nm diameter on average (see supplementary information, SI, for additional high-resolution TEM images). Additional chemical information is extracted from electron energy loss spectroscopy (EELS) and energy dispersive X-ray spectroscopy (EDXS). The latter reveal an overall composition of Co_3_FeC_0.25_O_2_, corresponding to individual contents of 64 at% metal, 32 at% oxygen, and 4 at% carbon. Such a small carbon content can in fact be caused by unavoidable deposition of amorphous carbon during the EDXS analysis. This is a consequence of electron beam induced deposition of hydrocarbons adsorbed or chemisorbed on the sample surface and from the residual gas. During deposition the main carbon source is the precursor which contains a substantial amount of carbonyl groups. For planar deposits we found that the carbon to oxygen ratio is close to one^28^. This indicates that carbonyl groups remain largely intact after dissociation and become part of the deposit, if their desorption is not sufficiently fast. Considering the small carbon content in the 3D structures in conjunction with the enhanced oxygen content, we argue that the oxygen is the result of a post-growth oxidation effect that occurred during sample transport and storage under ambient conditions before the TEM investigations were performed. Two further observations support this argument. First, scanning TEM EELS analysis of the oxygen content along the cross section of one of the nano-cube 3D edges reveals an enhanced oxygen content towards the surface (see 2(b)). However, as EELS signal intensities do not directly provide quantitative concentration data we have to deconvolve the signal. We therefore start from the following relation for quantitative elemental analysis (see, e.g.^30^).

1 $$I(\beta ,{\rm{\Delta }})=N{I}_{0}(\beta ,{\rm{\Delta }})\sigma (\beta ,{\rm{\Delta }})$$

for a given sample thickness, where *I* denotes the EELS intensity of the element of interest with areal density *N* in the energy range Δ beyond the element-specific threshold. *I*_0_ is the integral of the low-loss spectrum up to Δ, including the entire zero-loss peak, *σ*(*β*, Δ) is a partial cross section, and *β* is the collection semiangle. The O, Fe, and Co content may vary depending on the distance from the sample surface and the sample thickness will depend on the position of the EELS line scan. Tilt series via both, TEM and high-resolution SEM, reveal elongated instead of circular edge cross sections for 3D elements in agreement with previous studies^18,24^. To approach the real situation with a simplified analytical expression, we assume an elliptical cross-section of radii *R*_1_ and *R*_2_ for the two main axes of any of the nano-cube edges. From Eq. 1 we derive the following expression for the EELS intensity *I*(*β*, Δ; *x*) at any given position *x* along the line scan shown in Fig. 2(a) by integrating along the beam direction +*y* in the limits *y*_1_(*x*) and *y*_2_(*x*) defined by the respective sample thickness at scan position *x*

2 $$\begin{array}{rcl}\frac{I(\beta ,{\rm{\Delta }};x)}{{I}_{0}(\beta ,{\rm{\Delta }})\sigma (\beta ,{\rm{\Delta }})} & = & {\int }_{{y}_{1}(x)}^{{y}_{2}(x)}f(x,y){e}^{-y/{\rm{\Lambda }}(x,y)}dy\,{\rm{with}}\,f(x,y)\\  & = & {f}_{min}+({f}_{max}-{f}_{min}){e}^{-\xi (x,y)/\lambda }\end{array}$$

with

$${f}^{(O)}(x,y)={f}_{min}^{(O)}+({f}_{max}^{(O)}-{f}_{min}^{(O)}){e}^{-\xi (x,y)/\lambda }$$

for the oxygen distribution,

$${f}^{(Fe/Co)}(x,y)={f}_{max}^{(Fe/Co)}-({f}_{max}^{(Fe/Co)}-{f}_{min}^{(Fe/Co)}){e}^{-\xi (x,y)/\lambda }$$

for the iron and cobalt distribution and

$${\rm{\Lambda }}(x,y)={f}^{(O)}(x,y){{\rm{\Lambda }}}_{O}+\frac{{f}^{(Fe/Co)}(x,y)}{{f}^{(O)}(x,y)+{f}^{(Fe/Co)}(x,y)}\frac{3{{\rm{\Lambda }}}_{Co}+{{\rm{\Lambda }}}_{Fe}}{4}$$

with Λ_*O*_, Λ_*Fe*_ and Λ_*Co*_ denoting the electrons’ inelastic mean free path in O, Fe and Co, respectively (see “Methods” section for values). We assume here an exponentially decaying concentration of oxygen from the surface into the bulk, whereas in parallel the iron and cobalt concentrations are assumed to increase exponentially from the surface into the bulk with the same characteristic length *λ*. This simple concentration profile assumption is plausible, as a spinel phase may be concentrated at the surface (see electron diffraction analysis below), and yields good correspondence with the EELS line scan data (see below). *f*(*x*, *y*)*dy* represents the volume density or concentration of the respective element at point (*x*, *y*). *ξ*(*x*, *y*) is the vertical distance of the point (*x*, *y*) inside the cube edge from the surface. The result of fits using this model, assuming two different oxygen contents in the bulk (1 and 5 at%), is shown as solid lines in Fig. 2(b). The measured line scans can be reproduced quite well and clearly indicate a very low oxygen content (about 1 at%) in the center of the nano-cube edge that increases towards the surface to about 50 at% with a characteristic length of *λ* = 5 nm. At the same time, the metal content increases from about 50 at% at the surface to 99 at% in the bulk. Even a slight increase of the assumed oxygen content in the bulk to 5 at% leads to a distinctly different line profile of the fit that does not correspond well to the data. Due to the already high metal content, a change from 99 to 95 at% does not lead to a noticeable change of the corresponding fit. Given this, we assume a similar oxidation profile for the corresponding nano-cube and nano-tree structures used for the magnetic measurements.

A second, independent observation supports our assumption of a nearly 100 at% metal content in the bulk of our 3D nanostructures. Atom probe tomography on Fe- and Co-nano-pillars performed by Cordoba and collaborators showed that mass transport limited 3D growth provides favorable conditions for the complete desorption of carbonyl groups after dissociation^25^.

**Figure 3:**
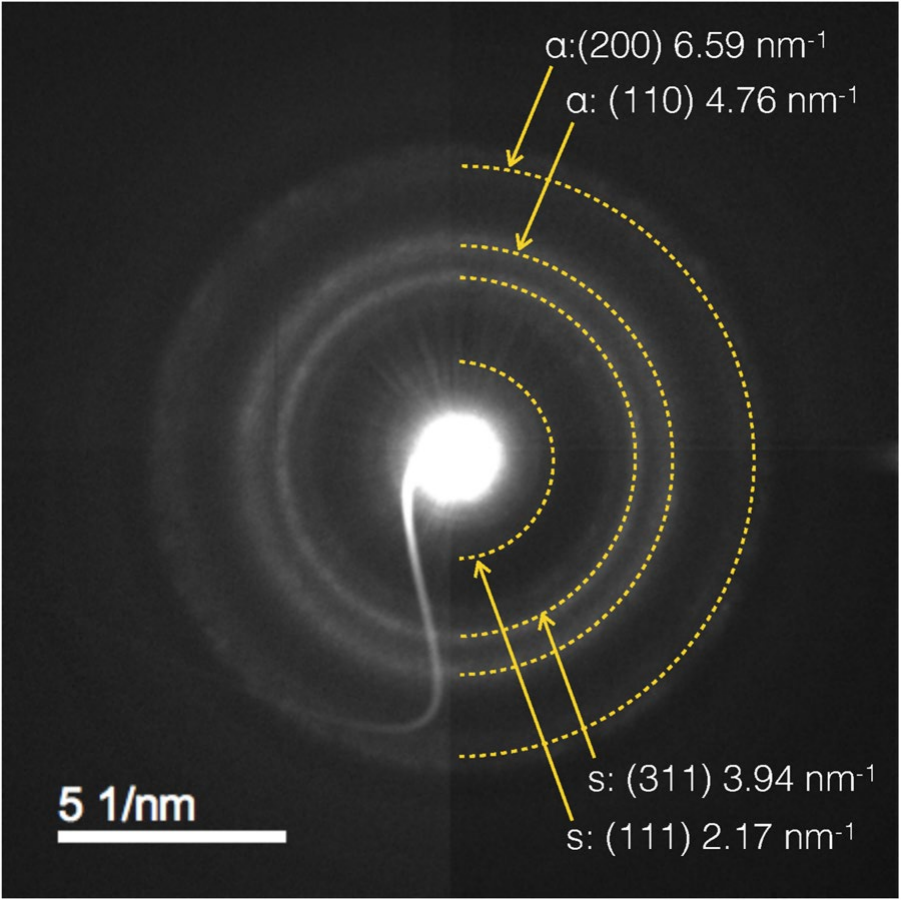
Electron diffraction. Selective-area electron diffraction of the region depicted by a green circle in Fig. 2(a). The dotted lines indicate the positions of the diffraction rings associated with the Co-Fe bcc phase (*α*) and the Co_2_FeO_4_ spinel phase (s).

In order to elucidate the degree of crystallinity of the deposits we performed selective area electron diffraction (SAED). The smallest field of view accessible in our setup is indicated in 2(a) by the shaded area (green circle). Consequently, we were not able to discriminate between the near-surface and bulk regions. This has to remain for future investigations. In Fig. 3 we show a diffraction image as measured and reference the diffraction rings with the corresponding scattering vectors. Apparently, the deposits are crystalline and we can attribute all diffraction rings to the *α*-phase of the Co-Fe binary system (bcc) and a Co-rich spinel phase of ferrimagnetic Co_2_ FeO_4_, which is expected to contain either amorphous or cubic cobalt-oxide phase contributions for metal ratio retention with regard to the precursor composition of Fe:Co = 1:3. This result corresponds well to our previous observations for planar Fe-Co deposits^28^.

Concluding this part on the microstructural characterization, we are led to consider the following microstructure concerning the magnetic properties of the deposits: a metallic *α*-Co_3_Fe core is surrounded by a metal-oxide sheath which is a ferrimagnetic spinel phase. Next we turn to the results of the magnetic measurements on the 2 × 2 arrays of nano-trees and nano-cubes.

### Micro-Hall magnetometry measurements and macro-spin simulations

**Figure 4:**
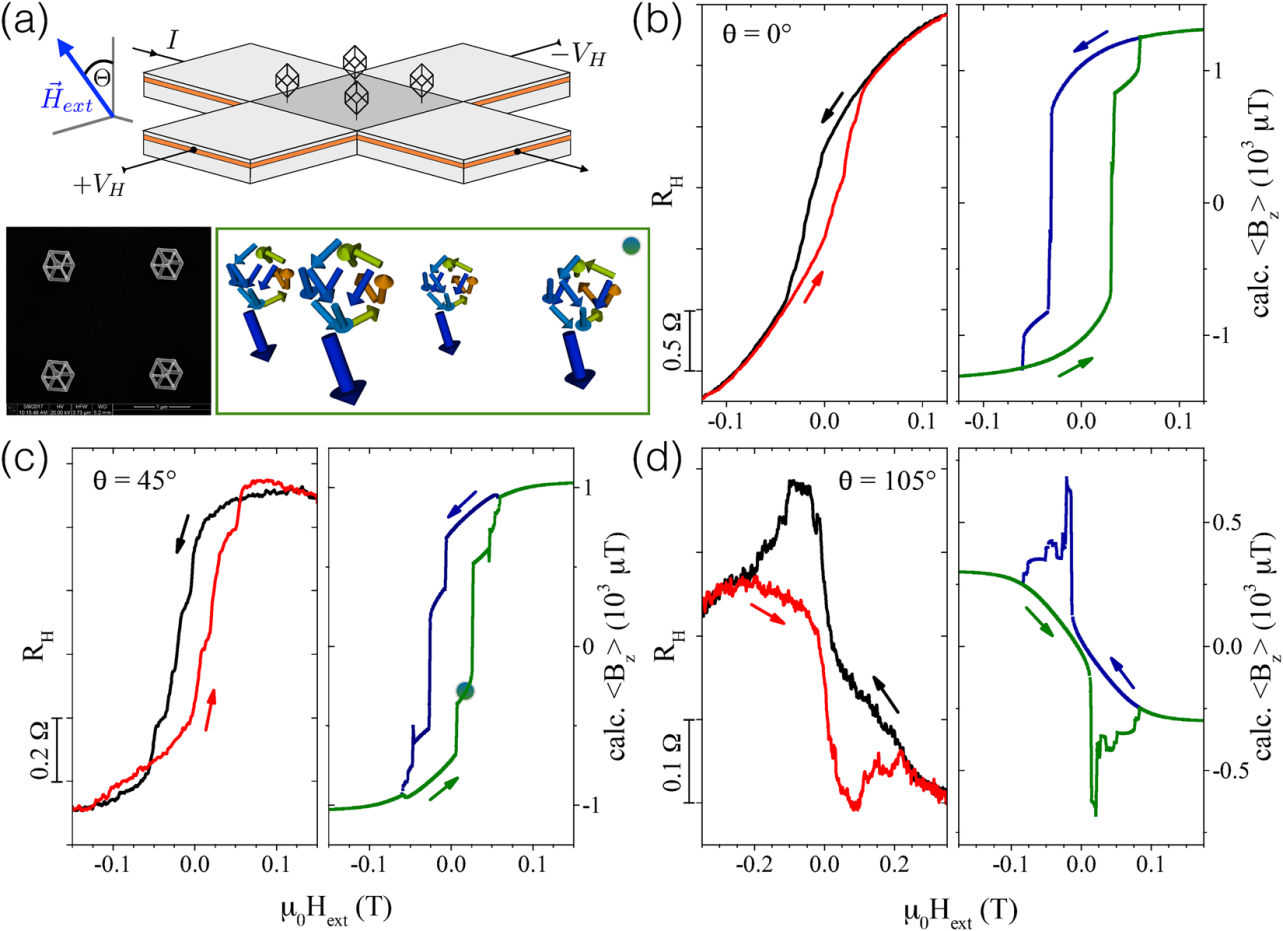
Micro-Hall magnetometry measurement results and macro-spin simulations of CoFe nano-cubes. (**a**) Sketch of a 2 × 2 array of CoFe nano-cubes on a 5 × 5 *μ*m^2^ Hall cross with the external magnetic field *H*_ext_ applied at an angle *θ* relative to the surface normal of the sensor (top). Bottom: SEM micrograph of the nano-cubes (top view) directly written by FEBID on top of the Hall sensor (left) and configuration of the magnetic moments from macro-spin simulations for *θ* = 45° (right) shown in (**c**). The circle marks the position at a positive field in the up-sweep, just before the switching of the stem. (**b**–**d**) magnetic hysteresis curves measured as *R*_*H*_ vs. *μ*_0_ *H*_e*xt*_ at *T* = 30 K compared to calculated stray fields from macro-spin simulations for *T* = 0 K for a selection of inclination angles *θ* equal to 0°, 45° and 105°, respectively. Arrows indicate the direction of field sweeps. Note different scales of the magnetic field axes in (**d**).

**Figure 5:**
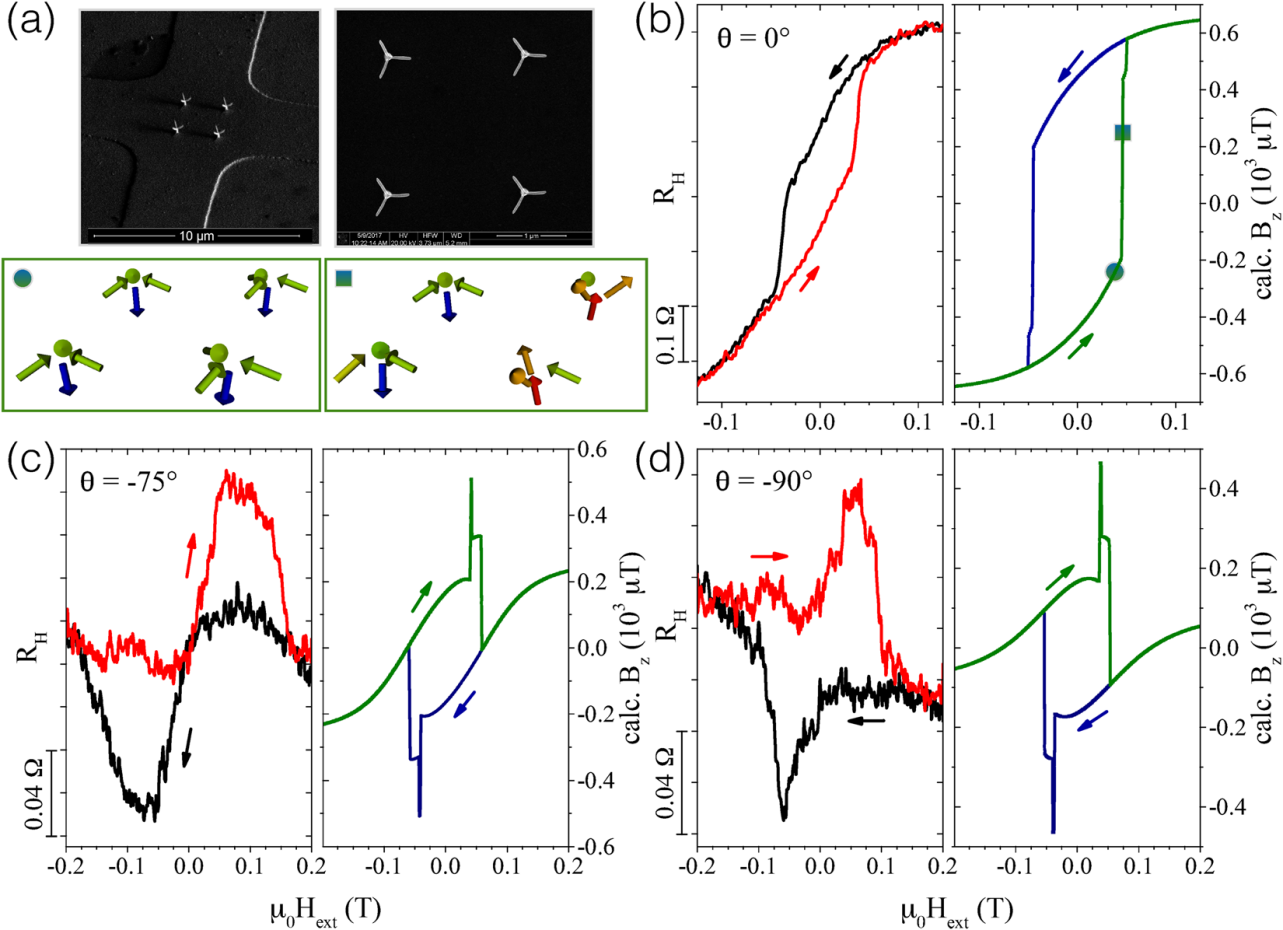
Micro-Hall magnetometry measurement results and macro-spin simulations for nano-trees. (**a**) Top: SEM micrograph of a 2 × 2 array of CoFe nano-trees on a 5 × 5 *μ*m^2^ Hall cross deposited by FEBID (left) and top view of the nano-trees (right). Bottom: Simulations showing the macro-spin configuration at *θ* = 0° for the up-sweep at two positive fields just before and at the switching of the stems’ magnetization marked by circle and square in (**b**). (**b**–**d**) Comparison of the magnetic hysteresis loops at *T* = 30 *K* and macro-spin calculations for *T* = 0 K and selected angles 0°, −75° and −90°, respectively. Arrows indicate the direction of field sweeps.

Competing magnetic interactions at the three- and four-edge vertices associated with nano-cube and nano-tree structures are expected to lead to non-trivial spatial magnetization profiles and, correspondingly, rather complex distributions of the magnetic stray field vectors. Micro-Hall magnetometry, as sketched in Fig. 4(a), is particularly well suited for measuring such stray fields of individual or small arrays of magnetic micro- and nanostructures^31–34^ in a wide range of temperatures and external magnetic fields applied under various angles with respect to the magnetic structures. In Figs 4 and 5 we show stray field measurements of the 2 × 2 nano-cube and nano-tree arrays for selected inclination angles of the external magnetic field (see Fig. 4(a) for the definition of the angle). The *z*-component of the stray field, 〈*B*_*z*_〉, emanating from the magnetic nanostructures is given by the measured Hall voltage difference Δ*V*_*H*_ = *α* ⋅ 〈*B*_*z*_〉 ⋅ *I*/*ne*, where *α* is a correction factor reflecting the Hall sensor’s response function which in turn depends on the electronic transport regime, *I* the applied current and *ne* the product of charge carrier density of the GaAs/AlGaAs Hall sensor and the electron charge. 〈…〉 denotes the average over the active area of the Hall cross and Δ*V*_*H*_ the *in situ* subtraction of the Hall signal of an empty reference cross in a gradiometry setup, see SI and methods section, where we also discuss the correction factor *α* in detail. Since *α* ≤ 1, we present the magnetic measurements as *R*_*H*_ ≡ *V*_*H*_/*I* ∝ 〈*B*_*z*_〉, which reflects the ratio of the nano-elements’ magnetization to the saturation value, *M*/*M*_*s*_.

We first discuss the magnetic nano-cubes. If applied along the *z*-axis (*θ* = 0°), the external field causes a magnetization reversal which appears to proceed rather continuously, see Fig. 4(b). A closer look, however, reveals step-like features in the stray field response. It is therefore instructive to compare the experimental stray field curves with a simple macro-spin approach, where stem and edges of the nano-cubes are represented by a single macro-spin based on the assumption that all microscopic magnetic moments point in the same direction and rotate collectively. Since such an approach omits the existence of shape anisotropy this has to be modeled by an additional uniaxial anisotropy for each macro-spin, see methods section for more details and the parameters used, see the SI for a discussion of the deviations between the measured and simulated absolute stray field signals. The model is lattice-based and mesh-free, which makes it very efficient for computing the mutual dipolar interactions of stems and edges within the nano-cube and -tree arrays. The almost vertical decrease or increase of 〈*B*_*z*_〉 of the simulated up- and down-sweep curves, respectively, at *θ* = 0° shown in the right panel of Fig. 4(b) are associated with the rotation of the four stems’ magnetization whereas the smaller steps are caused by the almost simultaneous flipping and canting of the edge macro-spins. Although the qualitative agreement is satisfying, the more rounded loop with smaller area and coercive field observed in the experiment points to a non-uniform magnetization switching of the stems dominated by multi-domain switching events.

A pronounced step-like switching behavior and better agreement of the measurements with the model is observed for a tilt angle of *θ* = +45° as shown in Fig. 4(c). Again, the large steps are associated with the rotation of the stems. Smaller steps and the finite slopes in between are connected with the flipping of edge spins and the rotation of their magnetization direction towards the external field. The closer the direction of the external field is to the anisotropy axis of an edge spin the larger is the coercive field resulting in the observed stair-case shape of the hysteresis. See SI for additional figures, which allow to assign the experimentally observed switching processes at *θ* = +45° and +105° to the nano-cubes’ magnetization states determined from the macro-spin simulations in the full magnetic field range.

A remarkable qualitative correspondence of the measured and simulated curves is seen for the complex and strongly pinched hysteresis loop at *θ* = +105° shown in Fig. 4(d), where *H*_*ext*_ is almost perpendicular to the anisotropy axis of the stems. Upon lowering the absolute value of the field, e.g. from negative saturation, all macro-spins relax towards their anisotropy axes. However, when approaching *H*_*ext*_ = 0, the edge spins are not in the lowest energy state but their total moment has a large component parallel to the external field axis whereas the stem spins point upwards precessing about their anisotropy axis. This remains the case even for small positive fields and causes the stem spins to suddenly rotate by 180° then pointing downwards. This causes the sharp peaks in the hysteresis which are observed in the measurements as well, however smeared out due to finite temperature and because the magnetization reversal mechanism of stem and edges are more complicated than the coherent rotation assumed in a single-domain macro-spin model. Nevertheless, although idealized, macro-spin simulations allow for identifying the relevant switching scenarios that occur for different inclination angles.

Next, we focus on the comparison of the measured nano-trees’ magnetization reversal with macro-spin simulations followed by a more sophisticated theoretical investigation based on micromagnetic simulations (see next section). As for the nano-cubes discussed above, the angular dependence of the measured stray fields, exemplarily shown for three angles *θ* = 0°, *θ* = −75° and *θ* = −90° shown in Fig. 5(b–d), respectively, demonstrates a rich variety of reversal processes determined by the field angle with respect to the stem and edges of the 3D nano-trees. If the external field *H*_*ext*_ is applied parallel to the stem (*θ* = 0°), the continuous progression of the stray field upon decreasing *H*_*ext*_ from saturation indicates a gradual rotational change of the magnetization direction followed by a switching process comprising a substantial part of the sample volume. The experimental hysteresis is qualitatively reproduced by the macro-spin model, which identifies the gradual change in magnetization with a simultaneous upwards canting and rotation of the edge macro-spins towards the direction of the applied field, while a fast switching sequence of the stems accounts for the sudden change of the magnetization, see the sequence of macro-spin configurations in the lower panel of Fig. 5(a) corresponding to two field values marked in the hysteresis loop of Fig. 5(b).

**Figure 6:**
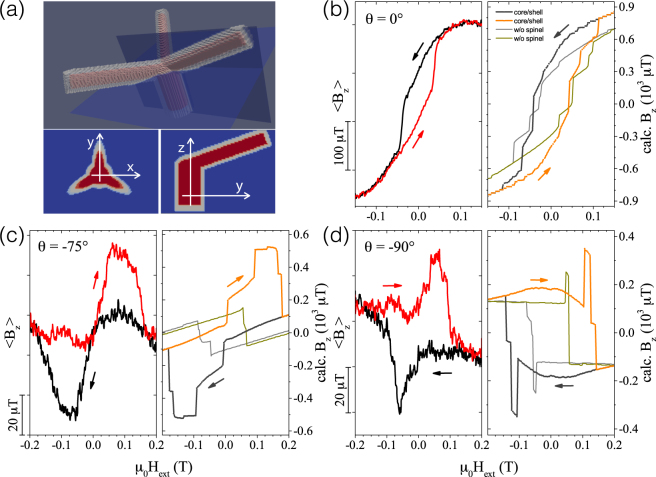
Micro-Hall magnetometry measurement results and micromagnetic simulations for nano-trees. (**a**) 3D-view and cross sections of nano-tree assuming a Co_3_Fe core (red) and Co_2_FeO_4_ spinel shell (gray), according to scenario 2 (core-shell structure). (**b**–**d**) Comparison of the magnetic hysteresis loops at *T* = 30 K and micromagnetic simulations for *T* = 0 K with or without core-shell structure (as indicated) for selected angles 0°, −75° and −90°, respectively. Arrows indicate the direction of field sweeps.

At an incident field angle of *θ* = −75° the field is nearly parallel to one of the edges. Here, a distinctly different magnetic hysteresis which narrows at zero applied field and exhibits a broad minimum and maximum at negative and positive fields for the down- and up-sweep curves, respectively, is observed. This behavior is only partly reproduced by the macro-spin model. The hysteresis loop shows maxima in the field cycle, but no narrowing of the loop occurs around zero field. Such a behavior requires a more finely grained effective spin model and is well reproduced by micromagnetic simulations considering a non-uniform reversal mechanism in the presence of the thin metal-oxide sheath, as shown in Fig. 6 below. In contrast, certain features of the stray field hysteresis for *θ* = −90° being less pinched and exhibiting sharper peaks are found in the macro-spin model. The pronounced peaks correspond to the rotation of the stems’ magnetization relative to the edges. At large fields, the magnetization of the edges and the stem are aligned along the field direction. Compensating stray fields from oblique magnetization angles of edges and stems lead to the small kinks near the peaks at smaller fields. A stronger compensation, which would reproduce the experimentally observed narrowing over a wide range of external fields at *θ* = −75° and *θ* = −90° is not found within the macro-spin model. Indeed, micromagnetic calculations, which are described in the next section, show a much more complex switching behavior beyond the limits of a fixed magnetic moment and a single axis anisotropy assumed in a macro-spin model.

### Micromagnetic simulations

Quite generally, micromagnetic simulations are invaluable for obtaining a deeper understanding of hysteresis effects by visualization of the magnetization reversal process on a microscopic scale^35^. At the same time, they are computationally much more demanding than macro-spin simulations, which still limits their application depending on the size of the simulation volume. However, using the parallel computing power of high-end graphics cards has led to about an order of magnitude faster code execution (see, e.g.^36^) and the development of multi-scale and multi-physics variants of micromagnetic solvers is already foreseeable^1,37^. Here we present results of micromagnetic simulations for the reversal process of the nano-trees in order to provide insight into the reasons for the discrepancy between the micro-Hall data and the macro-spin simulations. We also shed light onto the importance of taking the different magnetic behavior of the near-surface oxide into account. We focus on the nano-trees, because for these, the discrepancies compared to macro-spin simulations are most pronounced and they can still be treated by micromagnetic simulations if a core-shell structure consisting of metallic CoFe_3_ (core) and a ferrimagnetic spinel phase of Co_2_ FeO_4_ (shell) is assumed. Details on the simulation parameters are given in the methods section. For results of micromagnetic simulations of the nano-cubes we refer to the SI.

In Fig. 6(b–d) we present the results of the micromagnetic simulations at the same angles which have been shown before. We discuss two different material composition scenarios. The first scenario assumes an all-metal Co_3_Fe nano-tree, whereas the second scenario assumes an oxide shell of the ferrimagnetic spinel phase Co_2_FeO_4_ covering an all-metal Co_3_Fe core, as is schematically indicated in Fig. 6(a). For the spinel phase, the saturation magnetization is assumed to be a factor of 10 smaller than that of the Co_3_Fe core (see methods section for details where it is also explained how the average stray field was calculated). For the all-metal micromagnetic model we find the same qualitative behavior as in the macro-spin simulations. The overall correspondence with the micro-Hall data for *θ* = 0° is good and a reasonable agreement can be stated for −90°. This indicates that the macro-spin model catches the main features of the magnetization reversal processes in these cases, however see also the SI for a more detailed presentation of the spatial magnetization distribution as obtained from the micromagnetic simulations. In contrast to this, for *θ* = −75° the micro-Hall data are not reproduced by the all-metal micromagnetic or macro-spin simulations. However, if the core-shell structure with a spinel shell of reduced saturation magnetization is taken into account, we find for all angles a very good correspondence with the data. In particular, the pinched hysteresis form for *θ* = −75° is very well reproduced, and the shapes of the stray-field hysteresis for *θ* = 0° and *θ* = −90° are also very similar to the ones obtained by micro-Hall magnetometry. In addition, we observe that the coercive fields correspond quite well to the measured values. With regard to the remaining differences one has to take into account that our micromagnetic simulations assume 0 K, whereas the micro-Hall data shown here were taken at 30 K.

## Discussion

**Figure 7:**
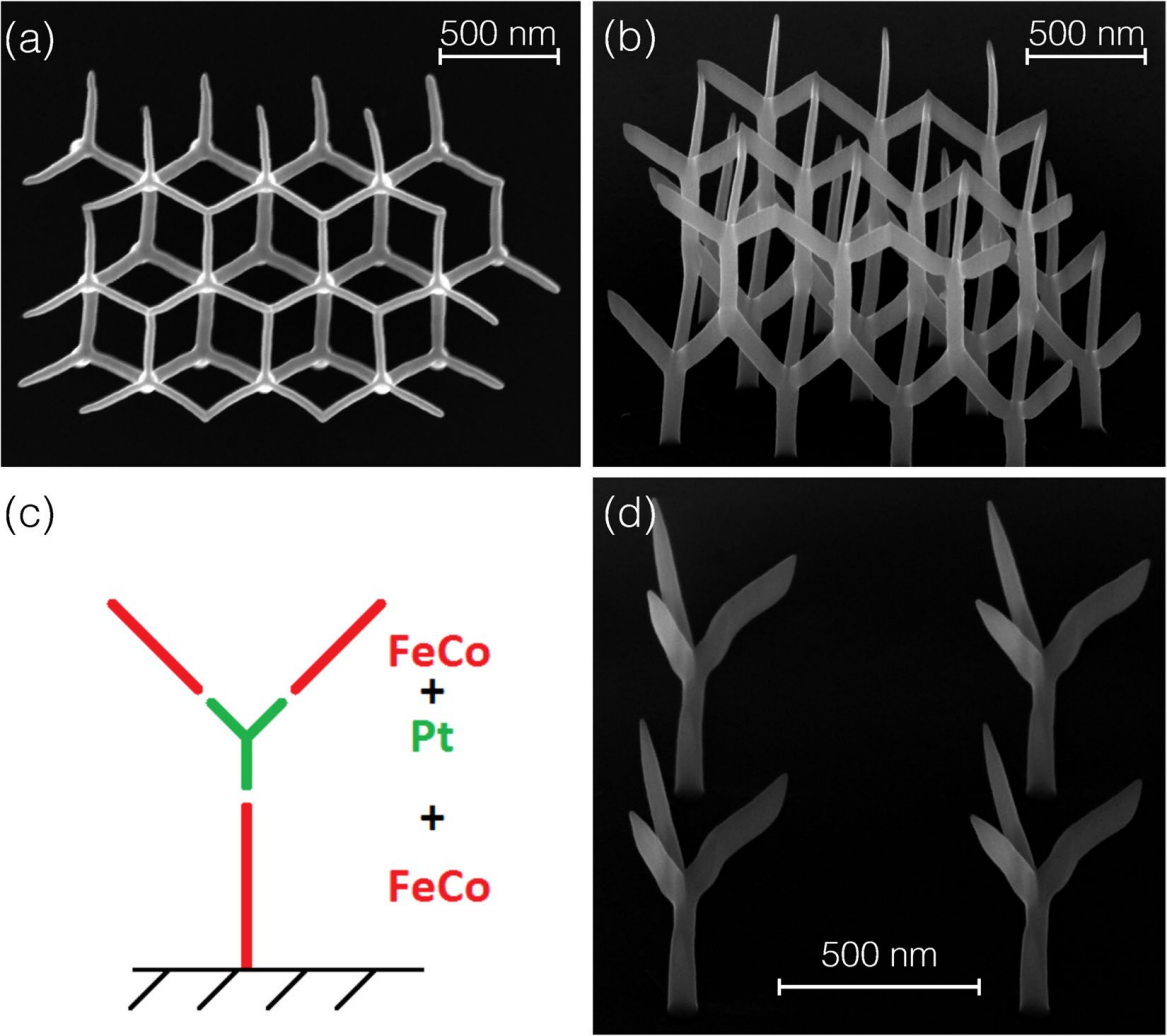
3D array of nano-trees and nano-tree with non-magnetic vertex. (**a**) SEM top view of 3D nano-tree array fabricated by FEBID with the precursor HCo_3_Fe(CO)_12_. (**b**) Tilted view of the same array. (**c**) Schematic of nano-tree for which the vertex part is replaced by a non-magnetic segment consisting of nano-granular platinum Pt(C). (**d**) SEM image of a 2 × 2-array of nano-trees with FeCo stems and edges and non-magnetic Pt(C) vertex segment.

We have shown that the main features of the magnetization switching of the 3D nano-architectures, as monitored by micro-Hall magnetometry, are already quite well reproduced by a fast and scalable macro-spin approach. If complemented by carefully designed micromagnetic simulations, the correspondence becomes very satisfying also in those cases for which the macro-spin model is less successful, see also^38^. In addition we note that from our micromagnetic simulations it becomes also quite apparent that for the presented case of interactions through a vertex, which is magnetic itself, the magnetization distribution inside the magnetic 3D structures can be rather complex (see SI for details). In view of a prospective application of the presented building blocks towards 3D artificial spin-ice systems it may be desirable to reduce this level of complexity in the magnetization distributions. For such arrays, micromagnetic simulations will not be feasible, and it will be exceedingly difficult to acquire a satisfying understanding of all details of the array’s switching behavior. Thus, we consider replacing the vertices in the nano-elements by non-magnetic material and demonstrate that the nano-elements can be arranged in 3D array structures by our FEBID approach. Figure 7 illustrates first results in these directions for the nano-tree geometry. Figure parts (a) and (b) show top and tilted views of a 3D nano-tree array employing again the precursor HCo_3_Fe(CO)_12_. In this case the FEBID writing strategy has to be carefully adapted to compensate for precursor gas flux shadowing effects^29^ and anisotropic growth or proximity bending of growing nano-elements^24^. If done properly, the nano-trees’ shape reproducibility and the placement accuracy are very good, as shown here. In figure parts (c) and (d) we demonstrate that it is furthermore possible to replace the vertex segment in the nano-tree by non-magnetic material, in our case nano-granular Pt, using Me_3_CpMePt(IV) as precursor.

## Conclusion

We have demonstrated that FEBID provides a powerful and flexible way to realize free-form magnetic 3D nano-elements and arrays of such structures. Our TEM-based characterization clearly indicates that HCo_3_Fe(CO)_12_ is a particularly well-suited precursor, as it leads to 3D deposits with nominally pure metallic character under beam conditions which are perfectly suitable for high-resolution structuring. The microstructure and composition analysis allowed us to pinpoint the essential features for suitable micromagnetic modeling (core-shell structure) and we were able to reproduce the most important stray field effects, as observed by micro-Hall magnetometry. In macro-spin model calculations, which are favorable because of their scaling behavior towards larger arrays, several observations in the switching behavior of the 3D nano-magnets can already be well reproduced. Replacing the vertex segment of the nano-elements by a non-magnetic material and the arrangement of nano-elements in 3D arrays was successfully demonstrated employing the FEBID approach. This will be of advantage for future work on 3D artificial spin-ice which is but one example of various other possible application fields of 3D magnetic FEBID structures on the single-element and array basis. Mesoscopic 3D arrays using this approach may allow for experimentally realizing and studying classical Ising or Heisenberg model systems^6^. A chiral geometry of the nano-elements in 3D arrays is of high interest with regard to magneto-optical properties (see, e. g.^39^). Finally, single 3D nano-magnetic elements with increasing geometrical complexity are conceivable for enhanced micro/nano-magnetic devices.

## Methods

### Febid

Samples were fabricated using a dual beam SEM/FIB (FEI, Nova NanoLab 600), equipped with a Schottky electron emitter operating at a base pressure of about 2 × 10^−7^ mbar. The precursors HCo_3_Fe(CO)_12_ and Me_3_CpMePt(IV) (Me: methyl, Cp: cyclopentadienyl) were injected in the SEM by means of a capillary with an inner diameter of 0.5 mm. The distance capillary-surface was about 100 *μ*m and the tilting angle of the injectors was 50°. The crucible temperature of the gas injection system (GIS) was set to 65°C and 45°C for the Co-Fe and Pt precursor, respectively. The electron beam parameters used during deposition were 20 keV for the acceleration voltage and 13 pA for the beam current. The dwell time was set after optimization of the 3D growth to 1 ms. The pitches depend on the inclination angle of the 3D structures and have to be adapted to the precursor and gas flow conditions^18^. Concerning the synthesis of the Co-Fe precursor we refer to^28^. As co-deposit or halo formation can have an influence on the switching behavior of magnetic FEBID structures, this has been discussed in some detail in the supplementary information. In the present case such an influence is very small.

### TEM

3D nano-cubes were deposited on Cu TEM grids. TEM investigations were carried out on a Tecnai F20 from FEI with a Schottky Field Emitter operating at 200 kV. Images were taken with a post-column energy filter (Gatan Imaging Filter, GIF) using an energy slit of 10 eV. The images were recorded zero-loss filtered (i. e., elastically scattered electrons only) on a 2 K charge coupled device. For the image recording and processing (Fourier transformation) the software DigitalMicrograph from Gatan was used. The EELS line profiles were taken at energy losses of 532 eV (O, K edge), 708 eV (Fe, L_3,2_ edge) and 779 eV (Co, *L*_3,2_ edge), respectively. The EELS measurements were done with 2.5 mm entrance aperture, a camera length of 100 mm, and a dispersion of 0.2 eV/ch for the spectrum. The exposure time (per pixel) was 3 s for the line scan (Spectrum Image). For the signal processing the background was subtracted using a power-law background model. The inelastic mean free paths used for the EELS line scan fits were Λ_*O*_ = 148.0 nm, Λ_*Co*_ = 110.4 nm and Λ_*Fe*_ = 111.5 nm, employing the standard calculation introduced by Malis *et al*.^40^.

### Micro-Hall magnetometry

The basic principle of operation of micro-Hall magnetometry is schematically sketched in Fig. 4(a). When a magnetic sample is placed on top of the sensor, the measured Hall voltage, *V*_*H*_, is proportional to the *z*-component of the sample’s magnetic stray field averaged over the active area of the Hall cross, 〈*B*_*z*_〉, via *V*_*H*_ = *α* ⋅ 1/*ne* ⋅ *I* ⋅ 〈*B*_*z*_〉. Here, *n* denotes the charge carrier concentration, *I* is the applied current, *e* the electron charge, and *α* a correction factor, which only in first approximation can be assumed *α* = 1. A detailed account of the correction factor and Hall response function related to the magnitude of the measured stray fields, as well as a discussion of background subtraction are given in the SI.

The homebuilt Hall sensor is fabricated from an MBE-grown AlGaAs/GaAs heterostructure hosting the two-dimensional electron gas (2DEG) as the sensitive layer. In a first step, standard UV-lithography followed by wet chemical etching is employed to form six adjacent Hall-crosses of 5 × 5 *μ*m^2^ nominal size. Then the sensor structure is electrically contacted by annealed AuGe/Ni contact pads and gold wire bonding. Subsequently the sensor is covered with a Cr/Au top-gate which also serves as substrate for the 3D nano-cubes and -trees directly written by FEBID, see the SEM micrograph shown in Fig. 1(b) in the SI. The gate is grounded during the measurements. The sensor can be operated in a wide magnetic field and temperature range, but is optimized for $4.2\,K\lesssim T\lesssim 100$ K.

After writing of the 3D magnetic nanostructures the Hall sensor has been transferred in less than one hour to a cryogenic system and Helium atmosphere equipped with a superconducting solenoid essentially free of magnetic flux jumps. The sample can be rotated with respect to the applied magnetic field, where *θ* = 0° for field angles perpendicular to the sensor plane.

### Macro-spin simulations

Each element of the nano-trees and nano-cubes is modeled by a single macro-spin. The shape anisotropy of each macro-spin is accounted for by a single uniaxial anisotropy. For the determination of the strength of this uniaxial anisotropy we assume that each edge and stem can be associated with a prolate ellipsoid. This allows us to calculate the anisotropy constant by means of the Stoner-Wohlfarth model^41^. Assuming an average magnetization of 1500 kA/m the corresponding uniaxial anisotropy values lead to coercive fields much higher than the experimental ones. Therefore, we have kept the calculated ratio between the stem and edge anisotropy of 1:1.3 and fitted the anisotropy constant to the experimental coercive field at an inclination angle of 0°. With a stem anisotropy constant of 285520 eV and a edge anisotropy constant of 336076 eV significant features of the experimental stray field hysteresis curves can be reproduced. This allows us to identify the underlying switching behavior of the different elements.

In order to study the dynamics of our macro-spin model we numerically solve the following damped spin dynamics equation (Landau-Lifshitz equation)

3 $$\frac{\partial {\overrightarrow{S}}_{i}}{\partial t}=-\gamma \frac{\partial H}{\partial {\overrightarrow{S}}_{i}}\times {\overrightarrow{S}}_{i}-\alpha \gamma (\frac{\partial H}{\partial {\overrightarrow{S}}_{i}}\times {\overrightarrow{S}}_{i})\times {\overrightarrow{S}}_{i}\,.$$

Eq. 3 describes the motion for a macro-spin ${\overrightarrow{S}}_{i}$ of unit length at site *i* caused by an effective field ${\overrightarrow{H}}_{{\rm{eff}}}$ which is generated by the interactions given in the Hamiltonian Eq. 4 below. In Eq. 3 *γ* denotes the gyromagnetic ratio and *α* a phenomenological damping factor in front of the so-called Landau-Lifshitz damping term that drives the macro-spin towards a full alignment with the effective field ${\overrightarrow{H}}_{{\rm{eff}}}$, i. e. a local or global minimum configuration. The macro-spin calculations are used to study the dynamic switching behavior of the nano elements. As the temperature of such non-equilibrium processes within a macro-spin model does not correspond to a physical temperature, we have performed the macro-spin simulations at *T* = 0 K.

The Hamiltonian describing the interactions among all macro-spins is given by

4 $$H=-\,D\sum _{i=1}{({\mu }_{i}{\overrightarrow{S}}_{i}\cdot {\overrightarrow{e}}_{i})}^{2}-\frac{{\mu }_{0}}{4\pi }\sum _{i < j}{\mu }_{i}{\mu }_{j}\frac{3({\overrightarrow{S}}_{i}\cdot {\overrightarrow{e}}_{i,j})({\overrightarrow{e}}_{i,j}\cdot {\overrightarrow{S}}_{j})-{\overrightarrow{S}}_{i}\cdot {\overrightarrow{S}}_{j}}{{r}_{i,j}^{3}}-{\overrightarrow{B}}_{{\rm{ext}}}\cdot \sum _{i=1}{\mu }_{i}{\overrightarrow{S}}_{i}$$

Here, the first term describes a uniaxial anisotropy, where *D* is the anisotropy constant and ${\overrightarrow{e}}_{i}$ is the unit vector pointing into the anisotropy direction. For *D* < 0 this term describes an easy-axis anisotropy. The second term is the dipole-dipole interaction, where *μ*_*i*_ describes the effective magnetic moment per macro-spin for the stem or the edge, respectively. The direction between two interacting macro-spins is given by the unit vector ${\overrightarrow{e}}_{i,j}$ and the distance is given by *r*_*ij*_. The last term in Eq. 4 is the Zeeman term which describes the interaction of the macro-spins with the external magnetic field ${\overrightarrow{B}}_{{\rm{ext}}}$.

We obtained our hysteresis curves by linearly ramping up and down the external field in the range of −0.2T ≤ *B*_ext_ ≤ 0.2 T using 10^6^ time steps of length Δ*t* = 10 ps. Using a damping constant of *α* = 0.3 such calculations are very fast and just need minutes on a single core of a common computer processor. The calculation of the stray fields 〈*B*_*z*_〉 was done in the following way. (1) The positions of the four nano-trees and nano-cubes on the Hall sensor area where determined from SEM images. (2) The cumulated stray field contributions of all macro-spins of the nano-tree/cube have been averaged over 420 × 420 positions in the *xy*-plane of the sensor array area (roughly 5 × 5 *μ*m^2^) at the *z*-position of the 2DEG 115 nm below the substrate surface.

### Micromagnetic simulations

Zero temperature micromagnetic simulations were performed by numerically solving Eq. 3 for a single nano-tree consisting of Co_3_Fe (scenario 1), Co_3_Fe/Co_2_FeO_4_ core/shell-structure (scenario 2) or a single nano-cube of Co_3_Fe (see SI for nano-cube). We used the GPU-accelerated micromagnetic simulation program MuMax3^36^ running on a Linux notebook with Intel Core i7-7700HQ processor, 32 GB random access memory and NVidia GeForce GTX 1060 graphics card. Using cubic voxels of edge length 5 nm for the finite difference discretization in MuMax3 the simulations for a typical external field cycle −0.2T ≤ *μ*_0_ *H*_ext_ ≤ 0.2 T at a step size of 0.0033T took about 60 hrs for one nano-tree. Simulations for the nano-cube with core/shell structure were not attempted, as the simulation volume and voxel number was expected to lead to simulation time of more than 260 hrs per external field cycle. Nano-cube simulations assuming full metallic Co_3_Fe as material were performed within 6 hrs for a typical field cycle. The simulation parameters were chosen as follows:

- **Nano-tree** Geometrical dimensions: stem diameter *D*_*s*_ = 119 nm (cylindrical) and length *L*_*s*_ = 185 nm, edge diameters *D*_*b*,1_ = 80 nm and *D*_*b*,2_ = 64 nm (elliptical) at a length of *L*_*b*_ = 340 nm, thickness of oxide spinel shell *t* = 2*λ* = 10 nm (scenario 2). Number of voxels in simulation volume: 124 × 106 × 66 = 867504. Material parameters: the saturation magnetization of the shell was set to ${M}_{S}^{({{\rm{Co}}}_{2}{{\rm{FeO}}}_{4})}=1.15\times {10}^{5}$ A/m using experimental data from^42^. For the Co_3_Fe-core we used ${M}_{S}^{({{\rm{Co}}}_{3}{\rm{Fe}})}=1.5\times {10}^{6}\,$ A/m and the exchange constant *A* = 1.4 × 10^−11^ J/m by averaging the respective value for Fe and Co^41,43^. As we could not find a reference for the exchange constant of the spinel we used the same exchange constant as for the core material.
- **Nano-cube** Geometrical dimensions: stem diameter *D*_*s*_ = 119 nm (cylindrical) and length *L*_*s*_ = 185 nm, edge diameters *D*_*b*_ = 62 nm (assumed cylindrical) at a length of *L*_*b*_ = 340 nm. Number of voxels in simulation volume: 102 × 118 × 148 = 1781328. Material parameters: the saturation magnetization was set to ${M}_{S}^{({{\rm{Co}}}_{3}{\rm{Fe}})}=1.5\times {10}^{6}$ A/m and the exchange constant to *A* = 1.4 × 10^−11^ J/m.

The nano-grain microstructure of the deposits leads to an averaging of the magnetic anisotropy, which is why we have omitted any anisotropy energy contributions in our simulations. In order to guarantee sufficiently fast convergence we set the damping parameter *α* to 0.3, used the full relaxation of MuMax3^36^ at the initial field value and then the conjugated gradient method for quicker convergence at all other field values of each cycle with a stop criterion of 10^−6^.

The simulation data on the orientation of the magnetic moment vectors within the volume elements of the nano-tree or nano-cube for each external field was used to calculate the stray field 〈*B*_*z*_〉 at the sensor layer in the following way. (1) The positions of the four nano-trees and nano-cubes on the Hall sensor area where determined from SEM images. (2) For each volume element of the nano-tree/cube the associated simulated magnetic moment was used to calculate the corresponding dipolar stray field. The stray field contributions of all moments of the nano-tree/cube set to one of the four positions were averaged over *n* × *n* positions of the sensor array area (roughly 5 × 5 *μ*m^2^) in the *xy*-plane at the *z*-position of the 2DEG 115 nm below the substrate surface. (3) The resulting four averaged stray fields were added to obtain the full averaged stray field of the four nano-trees/cubes. We checked that this coarse-grain averaging to obtain the overall stray field acting at the 2DEG position showed now appreciably changes anymore for *n* ≥ 28 which is why we set *n* = 28.

## Electronic supplementary material

Supplementary Information
