# Supplementary material for: Direct-write of free-form building blocks for artificial magnetic 3D lattices
Source: Sci Rep. 2018 Apr 18;8:6160. doi: 10.1038/s41598-018-24431-x (PMC5906635; doi:10.1038/s41598-018-24431-x)
Supplement: Supplementary file 1 — Supplementary Information [file 41598_2018_24431_MOESM1_ESM.pdf]

Supplementary information (SI)
Direct-write of free-form building blocks for artificial magnetic 3D lattices

Lukas Keller[1], Mohanad K. I. Al Mamoori[1], Jonathan Pieper[1], Christian Gspan[2], Irina Stockem[3], Christian Schröder[4], Sven Barth[5], Robert Winkler[2], Harald Plank[6], Merlin Pohlit[1], Jens Müller[1], and Michael Huth[1,*]

[1]Institute of Physics, Goethe University, Frankfurt am Main, Germany
[2]Graz Centre for Electron Microscopy, Graz, Austria
[3]Department of Physics, Chemistry, and Biology (IFM), Linköping University, Sweden
[4]Bielefeld Institute for Applied Materials Research, Bielefeld University of Applied Sciences, Bielefeld, Germany
[5]Vienna University of Technology, Institute of Materials Chemistry, Wien, Austria
[6]Institute for Electron Microscopy and Nanoanalysis, Graz University of Technology, Graz, Austria
[*]michael.huth@physik.uni-frankfurt.de

ABSTRACT

High-resolution TEM

The nano-granular microstructure of the 3D deposits becomes apparent in high-resolution bright field TEM images (see Fig. 1(a)). The grain size estimate is based on STEM-HAADF images, as shown in Fig. 1(b).

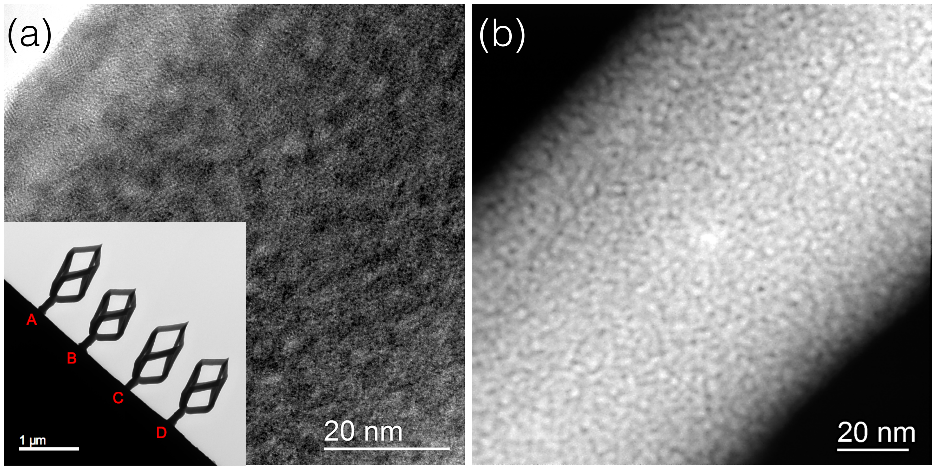

Figure 1. High-resolution TEM TEM bright field (a) and STEM-HAADF (b) image of a Co-Fe–based 3D branch, which allows combined, multiple measurements of the grain sizes to be found between 2.5 and 3.5 nm. The inset shows a low-magnification image of the analyzed nano-cubes.

Micro-Hall magnetometry

Principle of measurement

The magnetic stray field of a sample – which is directly linked to its magnetization[1] – is measured by detecting the Hall voltage V_H generated in the sensor plane formed by the 2DEG at the interface of the AlGaAs/GaAs heterostructure, see the schematics

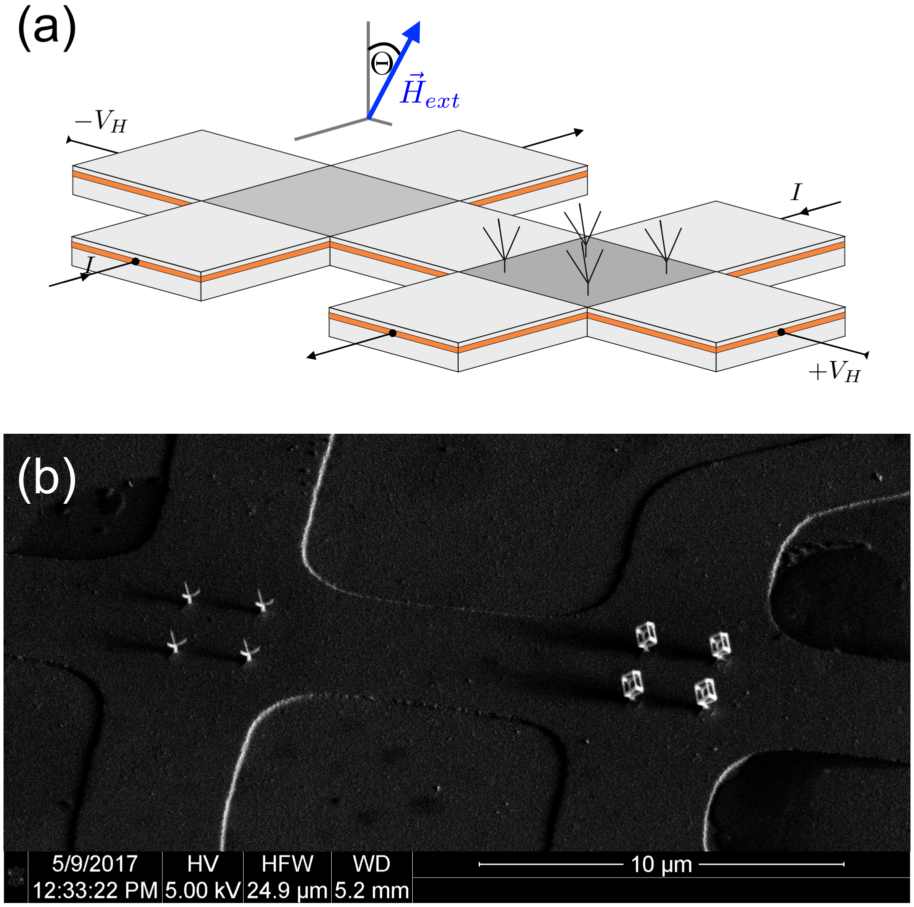

Figure 2. Micro-Hall gradiometry setup (a) Schematic of the Hall gradiometry technique, which allows for an *in situ* background correction by subtracting the signal which is linear in H_{ext} of an empty reference cross. The definition of the angle of the applied (b) SEM micrograph of the two adjacent Hall crosses accommodating 2×2 arrays of CoFe nano-trees and -cubes. The empty reference cross to the left is not shown.

in SI-Fig. 2(a). In first approximation, cf. Eqs. (1), (2) and (3), the detected z-component of the stray field averaged over the active area of the Hall-cross $\langle B_z \rangle$ is directly proportional to the measured Hall voltage V_H (see below for a detailed discussion).

Since the integrated stray field of the arrays of nano-cubes or -trees grown on top of the Hall sensor, see SI-Fig. 2(b), is more than two orders of magnitude smaller than the applied external field $\mu_0 H_{ext}$, a so-called gradiometry measurement is performed, where the large signal, which is linear in $\mu_0 H_{ext}$ is cancelled *in situ* by applying opposite currents across two Hall crosses, one decorated with magnetic particles and one empty, see Fig. 2(a). Then, the stray field contribution of the magnetic nanostructures is given by

$$\Delta V_H = \frac{1}{ne} \cdot I \cdot \langle B_z \rangle , \tag{1}$$

where $n = 3.4 \times 10^{11}\,\mathrm{cm}^{-2}$ denotes the carrier density of the sensor and I the applied currents, which have been $I = 2.5\,\mu\mathrm{A}$. The stray field $\langle B_z \rangle$ is detected in the active area of the Hall cross A in the plane of the 2DEG buried about 115 nm below the Cr/Au top-gate onto which the magnetic structures are grown.

Data of $R_H \equiv \Delta V_H / I \propto \langle B_z \rangle$ vs. $\mu_0 H_{ext}$ shown in this work have been corrected by subtracting a small linear background caused by slight differences between the two crosses in the gradiometry setup.

Stray field magnitude in measurement and simulation

Figures 4 and 5 of the main paper show the Hall magnetometry data as $R_H \equiv V_H/I$ vs. $\mu_0 H_{ext}$ implying that the simple equation (1) fails to describe (i) a Hall response function $F_H(x,y) \neq 1$ in the diffusive transport regime[2], where

$$V_H = \frac{1}{ne} \cdot I \cdot \frac{\int_A \mathrm{d}x\mathrm{d}y B_z(x,y) F_H(x,y)}{\int_A \mathrm{d}x\mathrm{d}y F_H(x,y)}, \tag{2}$$

and (ii) the influence of the inhomogeneous magnetic field distribution and the mean free path of the electrons in the 2DEG in the ballistic regime resulting in a correction factor α[3]:

$$V_H = \alpha \cdot \frac{1}{ne} \cdot I \cdot \langle B_z \rangle. \tag{3}$$

With the carrier concentration and mobility at $T = 30\,\mathrm{K}$ in the present experiment of $n = 3.4 \times 10^{11}\,\mathrm{cm}^{-2}$ and $\mu = 4.5 \times 10^5\,\mathrm{cm}^2/\mathrm{Vs}$, respectively, an effective mean free path of the electrons of $L_{\mathrm{eff}} \sim 4.3\,\mu\mathrm{m}$ can be estimated. This is still smaller but close to the structures size of nominally $5\,\mu\mathrm{m}$ (neglecting any edge depletion), therefore the transport may be at the crossover of quasi-ballistic to diffusive regime. For the latter, the correction factor (reflecting the response function $F_H(x,y)$) usually underestimates $\langle B_z \rangle$, i.e. $\alpha < 1$. For the former a general model is absent, which can lead to significant errors in the determination of the magnitude of the observed stray fields in absolute units[3]. Furthermore, it has been shown that α depends in a nontrivial way on the magnetic structure and the Hall cross geometry and may vary considerably, i.e., for quantitative magnetometry in non-diffusive systems, the Hall response must be calculated for each structure individually.

In our study, considering the increase of the Hall crosses' active area due to circular corners, the measured Hall signal becomes reduced by a factor of $\sim 2.5 - 3$. The estimated correction factor then varies between $\alpha \sim 0.45$ and 0.65 for different angles. These numbers are in agreement with values reported in the literature for the different transport regimes[2,3]. The variations of α with angle can be explained by the change in the magnetic field profile.

Despite these possible complications in determining the absolute stray field values, the measured Hall resistance $R_H \equiv V_H/I$ to a very good approximation is proportional to the actual stray field $\langle B_z \rangle$ averaged over the active area A of the Hall sensor, also under the condition that the responsive area is influenced by rounded corners and extends into the current and voltage leads. R_H reflects the ratio of the nanoelement's magnetization to its saturation value, M/M_s, in arbitrary units.

Macro-spin simulations

SI-Figures 3 and 4 show snapshots of the magnetic configurations of the CoFe nano-cubes during a hysteresis cycle in the full magnetic field range calculated by our macro-spin approach for $\theta = 45^\circ$ and $\theta = 105^\circ$, respectively. Only the configurations during the up-sweep of the external field are shown.

Comparison of micromagnetic and macro-spin simulations

SI-Figures 5 and 6 compare the micromagnetic (MM) and macro-spin (MS) simulations for a single CoFe nano-tree without and with the metal-oxide sheath consisting of a ferrimagnetic spinel phase.

The MS and MM simulations without the core/shell structure, shown in SI-Fig. 5, are very similar. One is therefore led to assume that the macro-spin model contains the essential features of the magnetization reversal process. However, the MM simulations show quite clearly that this assumption is premature. For the prominent stray field states (see solid- and dashed-line circles in the figure) selected, vortex-like magnetization profiles are visible at the terminal faces of the cylinder-shaped edges and also at the bottom of the stem. A closer inspection of different cross section through the nano-tree (not shown) reveals that these vortex structures are not threading throughout the full sample volume on any given cylindrical element. This is caused by the magnetic vertex segment joining the three edges and the stem of the nano-tree.

The situation is even more complex, if the nano-tree's core-shell structure is taken into account. In this case, the distribution of magnetization orientations seen in the MM simulations exhibits even stronger spatial inhomogeneities in the magnetization direction. At the end caps of the cylindrical edges hedgehog-like structures occur, whereas the pronounced vortex-like magnetization profiles observed for the all-metal nano-tree micromagnetic model are not as apparent anymore. We note, however, that the thickness of the spinel outer layer does only correspond to two voxel cells. One has therefore to be careful with regard to possible artefacts at strongly curved surfaces related to the finite-difference approach used in mumax3. The fact that the simulations predict a magnetization alignment perpendicular to the surface (see Fig. 6) is indicative that staircase discretization effects do indeed cause artefacts, see[4]. A more satisfying account on the details of the magnetization distribution in the shell would require a voxel edge length significantly below the used 5 nm. However, even at a moderate reduction to 3 nm the simulations could not be performed anymore on the hardware available to us (see method section of main text). Nevertheless, since the number of voxels representing the Co_3Fe core by far outnumbers the surface voxels, the obtained strayfield reversal curves should still give a valid account of the main features of the switching processes in the 3D nano-tree.

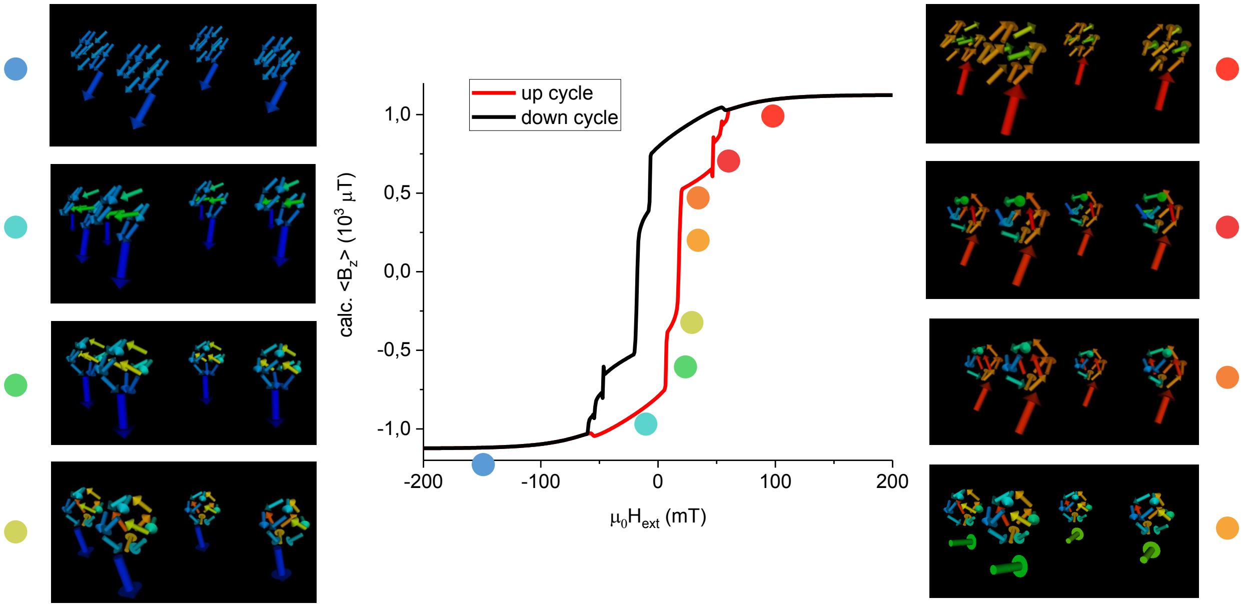

Figure 3. Magnetic configurations from macro-spin simulations of the CoFe nano-cubes during a hysteresis cycle for $\theta = 45°$. The large step is associated with the rotation of the stems. Smaller steps and the finite slopes in between are connected with the flipping of edge spins and the rotation of their magnetization direction towards the external field. The closer the direction of the external field is to the anisotropy axis of an edge spin, the larger is the coercive field resulting in the observed stair-case shape of the hysteresis.

Comparison of Hall magnetometry and micromagnetic simulations for 3D nano-cubes

In SI-Fig. 7 we show the results of micromagnetic simulations at the same angles which are shown in Fig. 4 of the main paper. The all-metal micromagnetic model already shows a very good qualitative agreement with the measured stray fields. For example, the experimentally observed crossing of the up- and down-sweep curves for $\theta = 45°$ are well reproduced by the simulations. As we have shown for the nano-trees, taking into account a core/shell structure with an outer metal-oxide layer we would expect an even better agreement with the measurements. However, for the nano-cube micromagnetic model the simulation volume is significantly larger than for the nano-tree. Even at the rather large 5 nm edge length for the cubic voxels used in our simulations, a core/shell structure simulation could not be performed anymore on the hardware available to us (see method section of main text).

Influence of co-deposit on magnetic switching behavior

From careful studies of the magnetic properties of planar Co nanostructures it is known that the co-deposit caused in FEBID by (mostly) backscattered electrons can influence the magnetic switching behavior, see e. g.[5,6]. It was furthermore shown that Xe or Ar ion beam milling can be effective in removing the co-deposit without causing excessive damage to the magnetic nanostructures proper[5,7].

In the present case co-deposit is mainly formed by secondary electrons generated by forward-scattered electrons exiting from the growing 3D structures and by secondary electrons generated by backscattered electrons from the early growth stages of the pillar-like stems which directly connect to the substrate surface. A substantial reduction of the co-deposit can be reached by operating under growth conditions in the electron limited regime, as in this case the contribution of transmitted electrons is minimized (but not completely eliminated). Our analysis (RW and HP) shows that for primary electron energies and beam currents of 20 keV and 13 pA, respectively, growth proceeds in the diffusion enhanced regime which is close to real electron limited conditions. By that, the number of "unconsumed" electrons transmitted through the 3D structures is not zero but small. From highly-resolved SEM images we estimate the continuous co-deposit thickness to be about 5 nm or less. However, this is very hard to accurately quantify without cross-sectional TEM studies and attempts to prepare such cross sections on the fine nano-tree or nano-cube structures are futile. We have therefore performed complimentary micromagnetic hysteresis calculations on a simple reference structure consisting of a Co_3Fe nano-pillar of 100 nm diameter and 500 nm height and compared the results with an analogous simulation for which in addition a disk-like oxidized Co-Fe layer of 600 nm diameter and a thickness

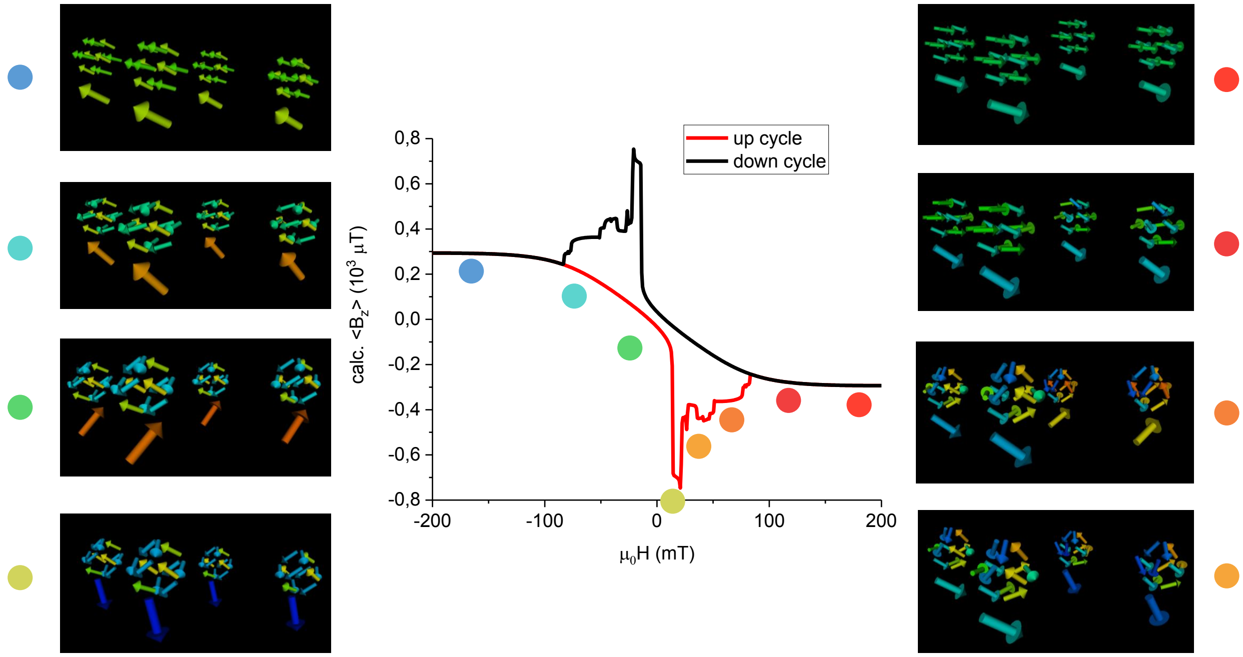

Figure 4. Magnetic configurations from macro-spin simulations of the CoFe nano-cubes during a hysteresis cycle for $\theta = 105°$. Note that H_{ext} is almost perpendicular to the anisotropy axis of the stems. Upon lowering the absolute value of the field, e.g. from negative saturation, all macro-spins relax towards their anisotropy axes. However, when approaching $H_{ext} = 0$, the edge spins are not in the lowest energy state but their total moment has a large component parallel to the external field axis whereas the stem spins point upwards precessing about their anisotropy axis. This remains the case even for small positive fields and causes the stem spins to suddenly rotate by 180° then pointing downwards. This causes the sharp peaks in the hysteresis which are observed in the measurements as well, however smeared out due to finite temperature and because the magnetization reversal mechanism of stem and edges are more complicated than the coherent rotation assumed in a single-domain macro-spin model.

of 10 nm was assumed (see insets of Fig. 8 for geometry). The simulation parameters were identical to those used in the micromagnetic simulations done for the nano-tree and nano-cube structures. Assuming a disk thickness of 10 nm represents a worst-case scenario for the influence of the co-deposit. We assume this disk to be oxidized, as is expected with a view to our TEM EELS results (see main text) and we assume that their metal content is the same as in the shell of the nano-trees or nano-cubes. In Fig. 8 we show the results for two external field orientations, namely parallel to the pillar axis (top row, $\parallel z$) and perpendicular to the pillar axis (bottom row, $\perp z$). In all cases we present the magnetization component perpendicular to the substrate surface, as this is detected by the Hall sensor.

For the parallel orientation the influence of the co-deposit within the model assumptions is to reduce the coercive field whereas the overall shape of the hysteresis is maintained. In perpendicular field orientation the influence of the co-deposit appears to be more pronounced. However, one has to take the M_z/M_S scale into account which is in both cases small. Nevertheless, the co-deposit causes an overall reduction of M_z by about 50 % and also influences the shape of the hysteresis.

What conclusions can be drawn from these model calculations with regard to the influence of co-deposit on the magnetic properties of the nano-tree and nano-cube structures? By way of inference, the overall good agreement of the shapes and characteristic switching fields of the stray-field hysteresis curves obtained by μ-Hall magnetometry in comparison with the results of the micromagnetic calculations for the nano-trees very clearly indicates that the co-deposit does not exert a strong influence on the switching behavior. This is most likely due to the fact that the overall magnetization of the co-deposit is smaller than was assumed in this worst case scenario calculation. This is in accordance with the general observation that the metal content in co-deposits tends to be substantially smaller than in the FEBID structures proper. Nevertheless, we consider it pertinent in future work to study whether ion beam milling for co-deposit removal can be done on these more complex structure without causing to much damage and whether this leads to significant changes in the magnetic switching behavior.

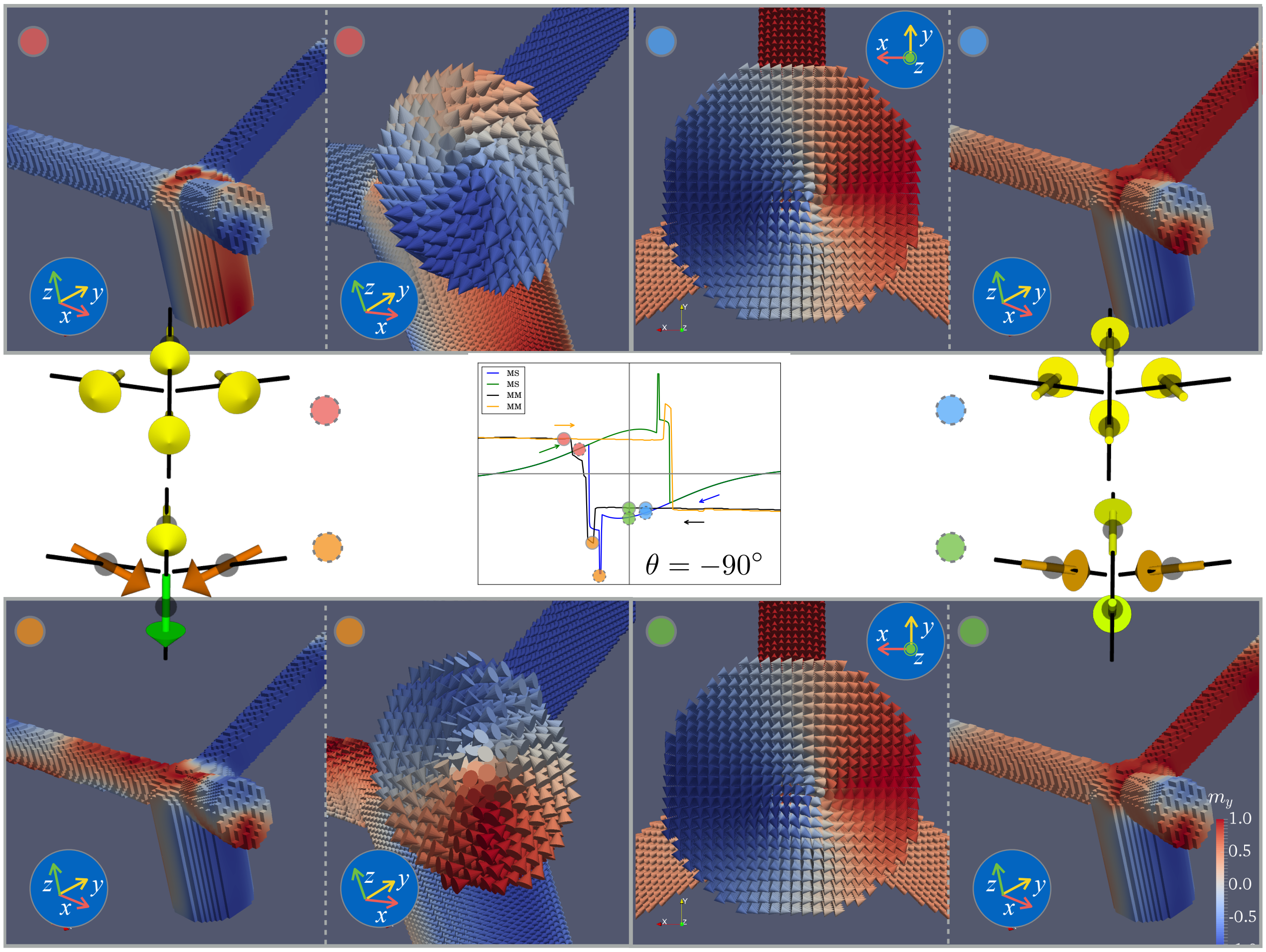

Figure 5. Comparison of macro-spin and micromagnetic simulations of a pure CoFe nano-tree. In the center the hysteresis loops for $\theta = -90^\circ$ are shown. Solid- and dashed-line circles mark the positions in the hysteresis loops where micromagnetic and macro-spin configurations, respectively, are shown. Arrows indicate the directions of the field sweeps. The color bar in the lower right sub-plot indicates the color-code for the magnetization's y-component (field direction): red – magnetization fully in field direction, blue – magnetization fully opposite to field direction. The same color coding is used for the sub-plots that show the respective magnetization direction by cones. For comparison, the macro-spins' orientations at the selected states indicated by the dashed-line circles are also shown. The colors of the macro-spins relate to the colors for the x-, y- and z-axis shown within the blue discs.

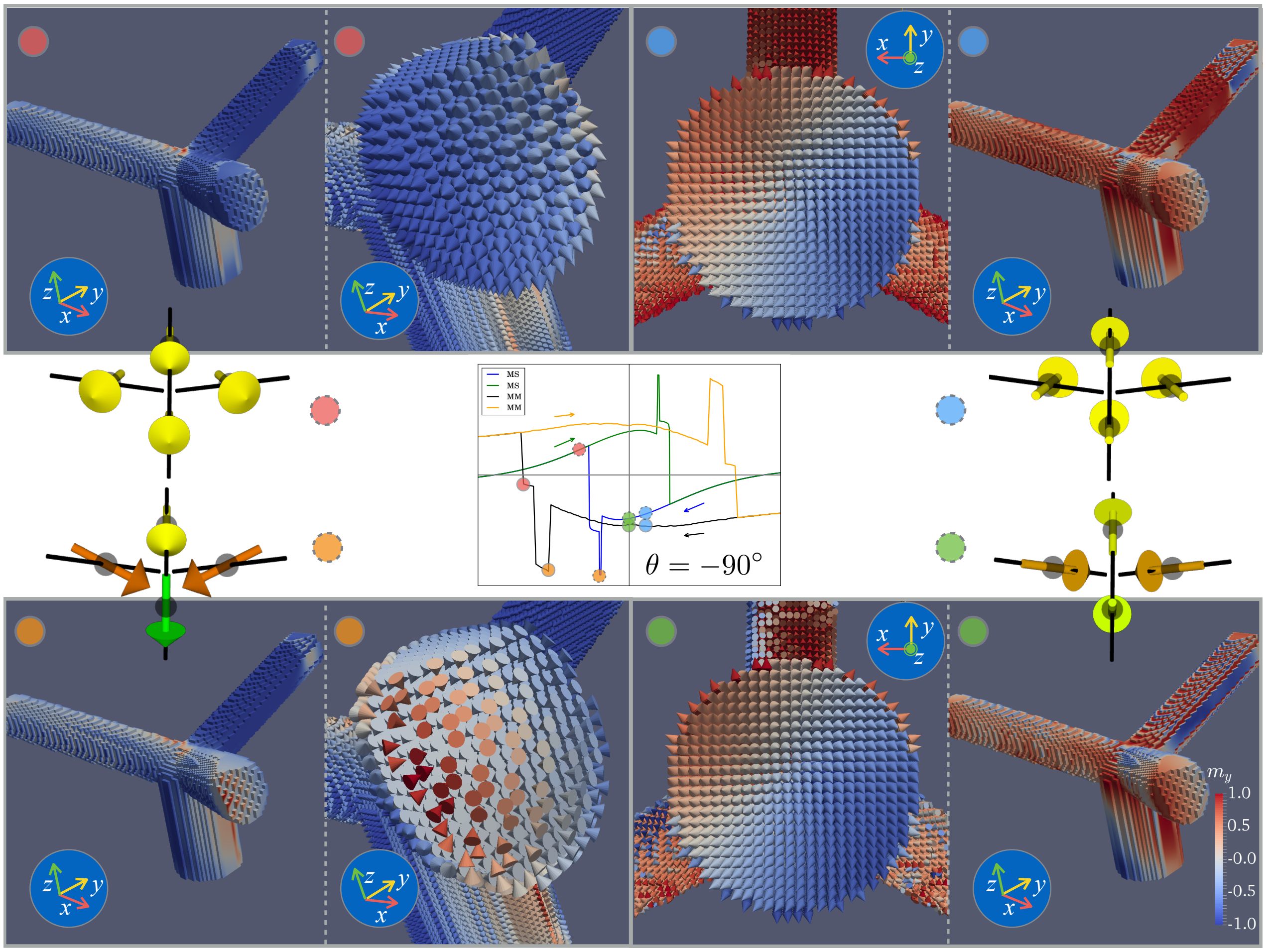

Figure 6. Comparison of macro-spin and micromagnetic simulations of a CoFe nano-tree with a metal-oxide ferrimagnetic shell. In the center the hysteresis loops for $\theta = -90°$ are shown. Solid- and dashed-line circles mark the positions in the hysteresis loops where micromagnetic and macro-spin configurations, respectively, are shown. Arrows indicate the directions of the field sweeps. The color bar in the lower right sub-plot indicates the color-code for the magnetization's y-component (field direction): red – magnetization fully in field direction, blue – magnetization fully opposite to field direction. The same color coding is used for the sub-plots that show the respective magnetization direction by cones. For comparison, the macro-spins' orientations at the selected states indicated by the dashed-line circles are also shown. The colors of the macro-spins relate to the colors for the x-, y- and z-axis shown within the blue discs.

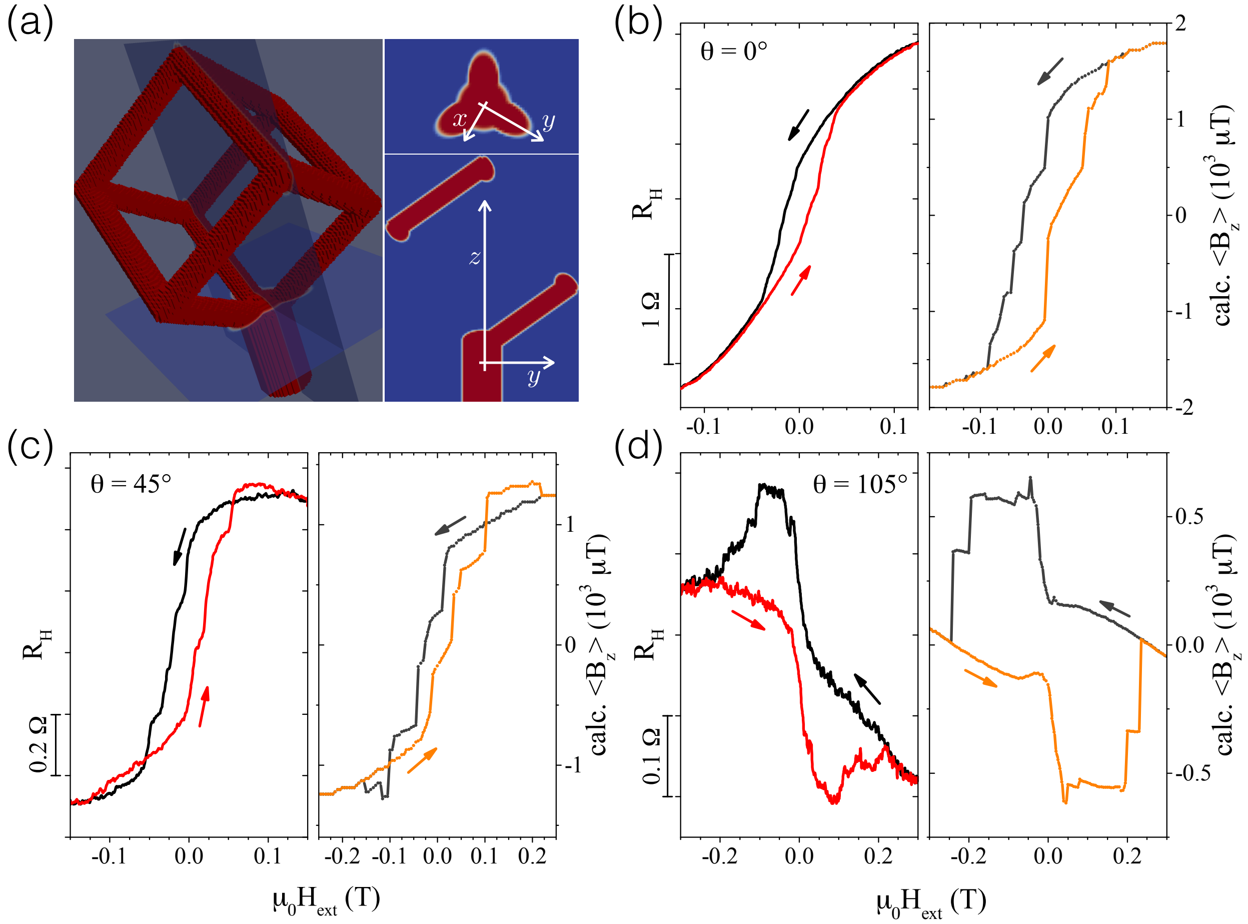

Figure 7. Comparison of Hall magnetometry and micromagnetic simulations of a all-metal Co_3Fe nano-cube (a) 3D-view and cross sections of nano-cube assuming a fully metallic Co_3Fe volume. The cross section planes are indicated in the 3D view. (b), (c) and (d) Comparison of the measured Hall resistance $R_H \propto \langle B_z \rangle$ at $T = 30\,\mathrm{K}$ and $\langle B_z \rangle$ calculated from micromagnetic simulations for $T = 0\,\mathrm{K}$ for selected angles 0°, 45° and 105°, respectively. Arrows indicate directions of field sweeps. Note different scales of the magnetic field axes in (b) and (c).

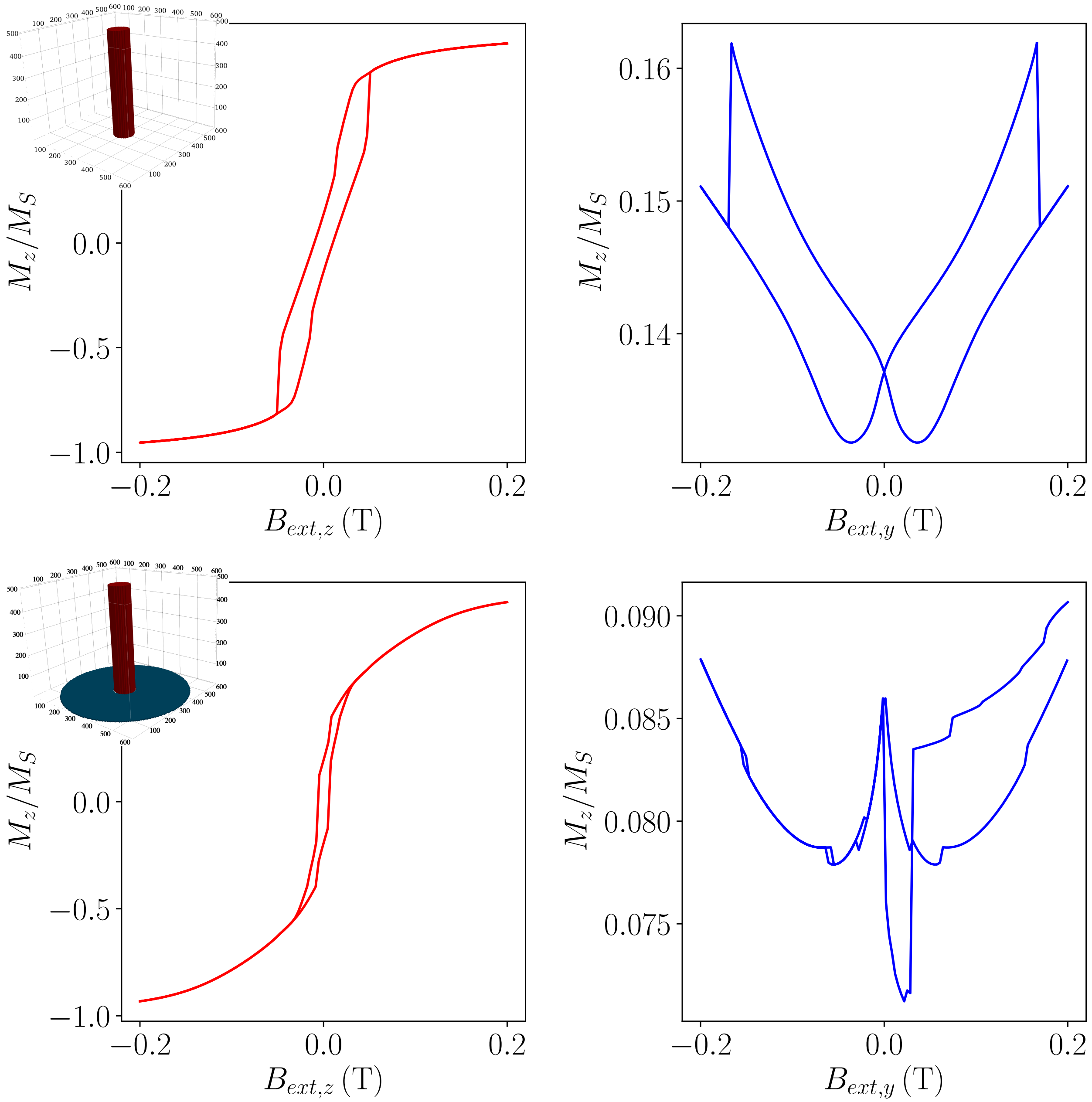

Figure 8. Micromagnetic simulation of Co_3Fe pillar with and without co-deposit. The upper row figures show results of micromagnetic simulations for a Co_3Fe nano-pillar with external magnetic field aligned parallel (left) and perpendicular (right) to the pillar axis. The lower row figures show analogous results if the pillar is in the center of a disk representing the co-deposit. For the disk the fully oxidized state of Co_3Fe is assumed.

References

1. Pohlit, M. et al., Experimental and theoretical investigation of the magnetization dynamics of an artifcial square spin ice cluster, *J. Appl. Phys.* **120**, 142103 (2016).

2. Cornelissens, Y.G. & Peeters, F.M., Response function of a Hall magnetosensor in the diffusive regime, *J. Appl. Phys.* **92**, 2006 (2002).

3. Cerchez, M. & Heinzel, T., Correction factor in nondiffusive Hall magnetometry, *Appl. Phys. Lett.* **98**, 232111 (2011).

4. García-Cervera, C. J., Gimbutas, Z. & Weinan, E., Accurate numerical methods for micromagnetics simulations with general geometries, *J. Comput. Phys.* **184**, 37 (2003).

5. Nikulina, E. at al., Origin and control of magnetic exchange coupling in between focused electron beam deposited cobalt nanostructures, *Appl. Phys. Lett.* **103**, 123112 (2013).

6. Fernández-Pacheco, A. et al., Magnetization reversal in individual cobalt micro- and nanowires grown by focused-electron-beam-induced-deposition, *Nanotechnology* **20**, 475704 (2009).

7. De Teresa, J.M. & Córdoba, R., Arrays of Densely Packed Isolated Nanowires by Focused Beam Induced Deposition Plus Ar^{+} Milling, *ACS Nano* **8**, 3788 (2014).
